# Biological Control of Chromium Redox and Stable Isotope Composition in the Surface Ocean

**DOI:** 10.1029/2019GB006397

**Published:** 2020-01-16

**Authors:** David J. Janssen, Jörg Rickli, Paul D. Quay, Angelicque E. White, Philipp Nasemann, Samuel L. Jaccard

**Affiliations:** ^1^ Institute of Geological Sciences and Oeschger Center for Climate Change Research University of Bern Bern Switzerland; ^2^ Institute of Geochemistry and Petrology ETH Zürich Zürich Switzerland; ^3^ School of Oceanography University of Washington Seattle WA USA; ^4^ School of Ocean and Earth Science and Technology University of Hawai'i at Mānoa Honolulu HI USA

**Keywords:** chromium, chromium redox, stable isotopes, biological productivity, paleoproxy, GEOTRACES

## Abstract

While chromium stable isotopes (δ^53^Cr) have received significant attention for their utility as a tracer of oxygen availability in the distant geological past, a mechanistic understanding of modern oceanic controls on Cr and δ^53^Cr is still lacking. Here we present total dissolved δ^53^Cr, concentrations of Cr (III) and total dissolved Cr, and net community productivity (NCP) from the North Pacific. Chromium concentrations show surface depletions in waters with elevated NCP, but not in lower productivity waters. Observed Cr deficits correspond well with calculated Cr export derived from NCP and Cr:C ratios of natural phytoplankton and marine particulates. Chromium (III) concentrations are stable over the diel cycle yet correlate with NCP, with maxima found in highly productive surface waters but not in lower productivity waters, indicating biological control on Cr (III). The relationship between Cr (III) and δ^53^Cr suggests that δ^53^Cr distributions may be controlled by the removal of isotopically light Cr (III) at an isotopic enrichment factor (∆^53^Cr) of −1.08‰ ± 0.25 relative to total dissolved δ^53^Cr, in agreement with the global δ^53^Cr‐Cr fractionation factor (−0.82‰ ± 0.05). No perturbation to δ^53^Cr, Cr, or Cr (III) is observed in oxygen‐depleted waters (~10 μmol/kg), suggesting no strong control by O_2_ availability, in agreement with other recent studies. Therefore, we propose that biological productivity is the primary control on Cr and δ^53^Cr in the modern ocean. Consequently, δ^53^Cr records in marine sediments may not faithfully record oxygen availability in the Late Quaternary. Instead, our data demonstrate that δ^53^Cr records may be a useful tracer for biological productivity.

## Key Points

- Chromium (III) correlates with biological productivity but not with irradiance, and no diel cycle is observed
- Biological export of Cr at previously observed Cr:C explains vertical [Cr] profiles locally and is similar to Cr sources globally
- Cr (III) export at Δ^53^Cr = −1.08‰ ± 0.25 relative to total dissolved δ^53^Cr matches our δ^53^Cr trends and the global δ^53^Cr‐[Cr] correlation

## 1 Introduction

Chromium (Cr) is primarily found as one of two redox species in natural waters: the toxic Cr (VI), which is largely inert to particle reactivity (e.g., Ellis et al., 2004), and the particle‐reactive Cr (III) (Cranston & Murray, 1978; Elderfield, 1970). Although thermodynamic predictions indicate that only Cr (VI) should be stable in oxygenated waters (Elderfield, 1970), both Cr (VI) and Cr (III) are found in the ocean (e.g., Achterberg & van den Berg, 1997; Connelly et al., 2006; Cranston & Murray, 1978; Jeandel & Minster, 1987), with a Cr (III) maximum in surface waters (e.g., Achterberg & van den Berg, 1997) due to either indirect photochemical and/or biological processes (Achterberg & van den Berg, 1997; Connelly et al., 2006; Kieber & Helz, 1992) or direct biological reduction (Li et al., 2009). The reduction of Cr (VI) to Cr (III) is accompanied by an isotope fractionation, resulting in an enrichment of light isotopes in the reduced Cr (III) species (e.g., Ellis et al., 2002), with overlapping fractionation factor ranges reported for different reductants (summarized in Wanner & Sonnenthal, 2013). Oxidative weathering and partial back‐reduction in riverine systems contributes isotopically heavy Cr to the ocean relative to bedrock sources (D'Arcy et al., 2016; Frei et al., 2014). Isotope fractionation associated with Cr reduction, along with the strongly different mobilities and reactivities of Cr (III) and Cr (VI), has led to the development of Cr stable isotopes (δ^53^Cr) as a useful proxy to reconstruct the oxygenation of the early earth (e.g., Frei et al., 2009; Frei et al., 2011; Planavsky et al., 2014) and suggests that redox cycling may control the distributions of dissolved Cr and δ^53^Cr in the modern ocean.

The first oceanic depth profiles of Cr identified a nutrient‐type distribution, with surface depletions and elevated concentrations at depth (Cranston & Murray, 1978; Campbell & Yeats, 1981; Cranston, 1983; Jeandel & Minster, 1984); however, this was much less clear than for other nutrient‐type elements (e.g., nitrogen, phosphorus, silicon, zinc, and cadmium; Nozaki, 1997, and references therein). Indeed, this biologically driven control, expressed both within depth profiles and as accumulation in deep waters along circulation paths, has been highlighted as fairly minor (Jeandel & Minster, 1987; van den Berg et al., 1994; Sirinawin et al., 2000; Connelly et al., 2006; Goring‐Harford et al., 2018; Rickli et al., 2019) and more recent studies have suggested that Cr may not completely follow nutrient‐type controls (e.g., Connelly et al., 2006; Moos & Boyle, 2019; Rickli et al., 2019; Sirinawin et al., 2000). Nevertheless, sediment trap data show a correlation between Cr and C in sinking particles (Connelly et al., 2006; Francois, 1988), and laboratory cultures of algal isolates indicate that Cr (III) is adsorbed onto and, to a lesser degree, incorporated into marine phytoplankton (Semeniuk et al., 2016), while both Cr (VI) adsorption (Ellis et al., 2004) and biological uptake (Semeniuk et al., 2016) are minor. Therefore, these observations support biological export involving Cr (III) species as a control on the marine Cr cycle, which as yet is poorly constrained.

Oxygen minimum zones (OMZs) in the ocean interior can influence Cr redox speciation (e.g., Murray et al., 1983; Rue et al., 1997), although this may be limited to anoxic environments, with data at present inconclusive regarding potential impacts in O_2_‐depleted yet not anoxic environments (Cranston, 1983). Clear and strong Cr redox changes, including removal of dissolved Cr, are observed in isolated local anoxic environments (Cranston & Murray, 1978; Emerson et al., 1979). However, anoxic environments in the modern ocean are mostly restricted to marginal seas and inlets (Breitburg et al., 2018), and available data from low‐O_2_ regions in the open and coastal ocean, including at or below O_2_ detection limits, do not support a large‐scale removal of Cr (Bruggmann et al., 2019; Cranston, 1983; Goring‐Harford et al., 2018; Moos & Boyle, 2019; Rue et al., 1997; Wang et al., 2019).

The first multibasin study of dissolved δ^53^Cr found a strong linear relationship between δ^53^Cr and ln[Cr] (Scheiderich et al., 2015), with an isotope enrichment factor (ε) of ≈ −0.80‰. Nearly all subsequent δ^53^Cr data from the open ocean fall on this trendline (Bruggmann, Klaebe, et al., 2019;Goring‐Harford et al., 2018 ; Moos & Boyle, 2019 ; Rickli et al., 2019), though coastal environments and basins with restricted circulation may be more susceptible to localized influences and may deviate from this relationship (Paulukat et al., 2016; Scheiderich et al., 2015). The global open ocean correlation suggests that one single process or a limited range of processes with similar fractionation factors act to control global ocean δ^53^Cr (Scheiderich et al., 2015). Based on isolated surface measurements from high productivity regions and available information on global ocean Cr cycling from earlier [Cr] studies, Scheiderich et al. (2015) proposed that this global correlation was the result of Cr reduction and removal in highly productive surface waters and in OMZs. However, data were not available to test either hypothesis at the time. Subsequent investigations of the role of OMZs in Cr removal and δ^53^Cr modification have indicated that these environments likely do not have a large impact (Bruggmann, Klaebe, et al., 2019; Goring‐Harford et al., 2018; Moos & Boyle, 2019; Wang et al., 2019). Similarly, while some recent δ^53^Cr data support the potential for biological removal of light Cr from surface waters (Moos & Boyle, 2019) and Cr export in high productivity shelf environments (Goring‐Harford et al., 2018), these and other studies have also highlighted that strong and clear support for the large‐scale impact of biological cycling on δ^53^Cr distributions is lacking (Goring‐Harford et al., 2018; Moos & Boyle, 2019; Rickli et al., 2019). Consequently, a mechanistic understanding of the factors behind the global δ^53^Cr‐ln[Cr] relationship remains limited.

In order to help advance this understanding, we present [Cr], [Cr (III)], and δ^53^Cr along with measurements of biological productivity in the North Pacific. These parameters, which have been measured together for the first time in this study, can help to assess the role of biological productivity and export on [Cr], Cr redox speciation, and δ^53^Cr.

## 2 Methods

### 2.1 Study Area

**Figure 1:**
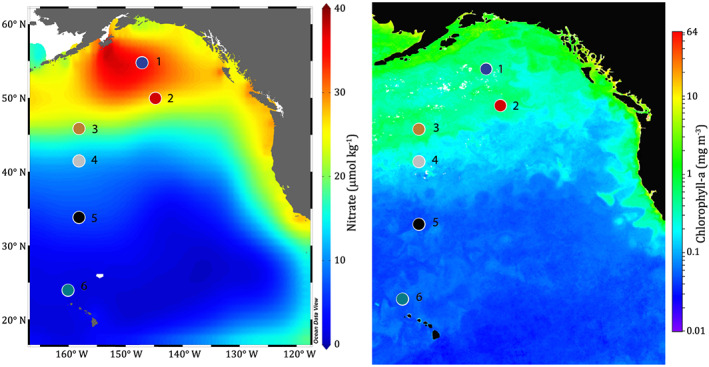
Map of stations with surface NO_3_^‐^ and chlorophyll *a*. Stations are numbered, with colors corresponding to those used in subsequent figures. Nitrate data are annual average values from the World Ocean Atlas (Garcia et al., 2014). Chlorophyll data are from satellite observations (Visible and Infrared Imager/Radiometer Suite Suomi National Polar‐orbiting Partnership [VIIRS SNPP]) from September 2017, the same month as the voyage (National Oceanic and Atmospheric Administration [NOAA] Star Ocean Color, National Environmental Satellite, Data, and Information Service [NESDIS] Center for Satellite Applications and Research, http://www.star.nesdis.noaa.gov/sod/mecb/color/).

Samples were collected at six stations on board *RV Kilo Moana* cruise KM1713 from Seward, Alaska, to Honolulu, Hawai'i (Figure 1), from the subarctic to the subtropical North Pacific. The eastern subarctic North Pacific consists of the Alaskan Gyre, an upwelling gyre characterized by high surface macronutrient concentrations and chronic Fe limitation (e.g., Boyd & Harrison, 1999). This region is characterized by relatively stable biological productivity on seasonal and interannual timescales, with a phytoplankton community dominated by small cells (<5 μm) composed primarily of autotrophic flagellates, small diatoms, and *Synechococcus* (Boyd & Harrison, 1999). Winter mixing in the eastern subarctic North Pacific is limited to roughly 100 m due to a strong and permanent halocline (Freeland et al., 1997).

The subtropical North Pacific consists of the large oligotrophic North Pacific gyre, with biological productivity controlled by very low concentrations of macronutrients in surface waters and by the seasonal penetration of light to greater depths (Letelier et al., 2004). Chlorophyll concentrations vary on seasonal and interannual cycles, and the biological community in dominated by small cells (<5 μm), of which *Prochlorococcus* is the primary contributor (Letelier et al., 1993). Maximum winter mixing depths reach approximately 90 m (Letelier et al., 2004).

The transition zone between these two gyres is a highly productive and dynamic environment, which moves latitudinally throughout the season (Ayers & Lozier, 2010; Juranek et al., 2012). Winter mixing in this region extends to 100–150 m; however, lateral advection dominates macronutrient supply (Ayers & Lozier, 2010). The phytoplankton community reflects the transition between the subtropical (*Prochlorococcus*‐dominated) and subarctic (primarily *Synechococcus* and small diatoms) regimes, as well as an elevated abundance of coccolithophores (Juranek et al., 2012).

These contrasting systems contain some of the longest‐running oceanographic time series: the Line P transect time series (Line P, Ocean Station PAPA/P26; our station 2) in the subarctic North Pacific and the Hawaii Ocean Time Series (HOT; our station 6) in the subtropical North Pacific. Therefore, this expedition across differing nutrient regimes and gradients of biological productivity provides dynamic and well‐studied environments on which to build our understanding of Cr cycling. Previous studies at Station P26 have reported a depth profile for [Cr] and [Cr (III)] (Mugo & Orians, 1993) and [Cr] and δ^53^Cr at 10 and 1,000 (Scheiderich et al., 2015).

### 2.2 Seawater Sampling

Samples were collected as part of a pseudo‐Langrangian investigation of primary productivity. Once on station (stations 2–5), a profiling float was deployed that was programmed to cycle in the upper 200 m and to surface roughly every 2.5 hr. The ship followed this float for at least two full diel cycles, with CTD‐rosette casts every 2 hr. Samples for Cr analyses were collected every 4–8 hr for two diel cycles, giving replicate samples throughout time. The profiling float was not deployed at station 1, and instead two diel cycles were followed at a fixed location. Samples were collected for only one diel cycle at station 6.

Seawater samples were collected in 12‐L Niskin bottles on a conventional rosette. Samples were gravity filtered through 0.22‐μm Acropak capsule filters (Pall Corporation) into acid‐cleaned 1‐L low‐density polyethylene bottles ([Cr] and δ^53^Cr) and acid‐cleaned 15‐ml polypropylene centrifuge tubes ([Cr (III)]). Chromium (III) samples were processed at sea. One‐liter samples were acidified with 2 ml 12 M HCl (sub‐boiling distilled) on land and stored for at least four additional months before processing and analysis, resulting in complete conversion of all Cr to Cr (III) (Semeniuk et al., 2016).

### 2.3 Shipboard Cr (III) Processing

Filtered samples were visually adjusted to the 15‐ml graduation on the centrifuge tubes, and Cr (III) was isolated using magnesium hydroxide (Mg (OH)_2_) coprecipitation (Semeniuk et al., 2016) by the addition of 60‐μl saturated NH_4_OH. The NH_4_OH solution was prepared by bubbling NH_3_ gas (Praxair) into 18.2 MΩ water. Precipitation was typically initiated within 15 min of sample collection, and at most within 2 hr of collection. After the formation of a precipitate, the vials were shaken, left for 10 min, shaken again, and centrifuged for 5 min. The supernatant was carefully decanted, and samples were dissolved in 1 ml 1% HNO_3_ (baseline, Seastar Chemicals).

While the Mg (OH)_2_ coprecipitation method has been shown to give quantitative recovery of Cr (III) (Semeniuk et al., 2016), there remains uncertainty if coprecipitation methods can recover organically complexed Cr (III) or only reactive Cr (III) species at and above native seawater pH (e.g., Nakayama et al., 1981). Nevertheless, if some fraction of strongly complexed dissolved Cr (III) exists that is not recovered by adsorption onto Mg (OH)_2_, this fraction would presumably be inert to reaction with other particles in seawater. Therefore, whether or not Mg (OH)_2_ yields quantitative recovery of total dissolved Cr (III), Mg (OH)_2_‐reactive Cr (III) species are likely the relevant species for discussing other Cr (III) adsorption or uptake processes. Here we refer to the reactive, measured Cr (III) species as Cr (III) and any putative not‐recovered Cr (III) species as Cr (III)_NR_.

### 2.4 Sample Purification for [Cr] and [Cr (III)]

Sample preparation for [Cr] is described in detail in Rickli et al. (2019). In brief, 30‐ml aliquots were taken from acidified 1‐L δ^53^Cr samples, spiked with ^50^Cr and left to equilibrate overnight. Chromium was then enriched from seawater by Mg (OH)_2_ coprecipitation and purified by cation exchange chromatography using AG 50W‐X8 resin (Bio Rad; see also Yamakawa et al., 2009). Shipboard‐enriched Cr (III) samples were spiked with ^50^Cr and carried through the same cation column procedure as [Cr] samples. The associated chromatography blank was 10–20 pg Cr, and the reagent blank for [Cr (III)] precipitation was 18 pg Cr. Chromium (III) concentrations have been corrected for blank contributions. Total [Cr] obtained on 30‐ml subsamples were used for spiking purposes only.

### 2.5 δ^53^Cr Sample Purification

Chromium separation for δ^53^Cr and [Cr] on 1‐L samples followed procedures described thoroughly in Rickli et al. (2019). Briefly, 1‐L samples were spiked with a ^50^Cr‐^54^Cr double spike and left to equilibrate for at least 24 hr. Chromium was enriched from seawater by Mg (OH)_2_ coprecipitation (see also Moos & Boyle, 2019) and processed through two sets of ion exchange chromatography—(1) anion chromatography using AG 1‐X8 resin following Cr oxidation with ammonium persulfate and with reductive Cr elution (see also Ball & Bassett, 2000; Moos & Boyle, 2019) and (2) cation chromatography using AG 50W‐X8 resin (see also Yamakawa et al., 2009). Initial samples for this study were oxidized with 0.2 M ammonium persulfate (TCI chemicals), which accounted for the majority of the blank (Rickli et al., 2019). Subsequently, it was determined that 0.1 M ammonium persulfate was sufficient for sample oxidation. Therefore, blanks ranged from those reported in Rickli et al. (2019); ~14 pmol Cr) to ~7 pmol Cr when 0.1 M ammonium persulfate was used. Blanks were 2 orders of magnitude below sample Cr and therefore did not affect data quality.

### 2.6 Mass Spectrometric Analysis of [Cr], [Cr (III)], and δ^53^Cr

Chromium samples were analyzed on a Neptune Plus MC‐ICP‐MS (Thermo Scientific). Redox and preliminary total Cr concentration data were obtained using the isotope dilution methodology described in Rickli et al. (2019). An average [Cr (III)] with 1 standard deviation (*SD*) error is presented in tables and figures for depths that were replicated across different points in time. For nonreplicated depths, a conservative total combined uncertainty of 10% is applied, of which the initial sample volume is the largest component. Internal analytical uncertainties from the measurements were less than 1%.

Chromium isotope composition is expressed as the deviation of the ^53^Cr/^52^Cr ratio relative to NIST SRM 979 in δ notation (equation (1)).

(1) δ53Cr000=5352CrSample5352CrNISTSRM979−1×1,000

The mass spectrometric procedures for the measurement of δ^53^Cr and final [Cr] are described in detail in Rickli et al. (2019). In brief, ^50^Cr, ^52^Cr, ^53^Cr, and ^54^Cr were monitored along with the masses 49, 51, and 56 for isobaric interference corrections of Ti, V, and Fe, respectively, on Cr. The most significant isobaric interference was from ^54^Fe on ^54^Cr, contributing a maximal uncertainty of 0.005‰, roughly an order of magnitude smaller than our external reproducibility of 0.02–0.03‰ (2 *SD*, see below).

Raw data were corrected offline following Rudge et al. (2009) and adjusted to the daily mean δ^53^Cr of double spiked NIST SRM 979 (*N* ≥ 11). Internal errors were 0.02–0.03‰ (2 standard error of the mean, SEM) for 100‐ppb solutions, the target concentration for analysis. The external reproducibility was 0.021‰ (2 *SD*; *N* = 77; Figure S1 in the supporting information) for 100 ppb NIST SRM 979 when corrected for the daily average NIST value following Schoenberg et al. (2008) and 0.033‰ (2 *SD*) for replicate analyses of seawater standards and samples following Kenney and Keeping (1951); *N* = 49 analyses of 17 seawater samples, including replicates and intercalibration samples from Rickli et al., 2019; Table S2 in the supporting information). The external reproducibility for [Cr] is 0.82% (1 relative standard deviation (RSD); *N* = 49 analyses of 17 samples/seawater standards). Replicate isotope measurements from different time points (*N* = 2‐3) are presented as weighted means with 2 SEM, and individual isotope measurements are presented in figures with either 2 *SD* external reproducibility estimated from replicates of processed seawater or with 2 SEM internal error, whichever is larger. All field data are shown in Table S1. A laboratory intercomparison was conducted at the same time as this study, confirming intercomparability of our data with previous studies and is presented in Rickli et al. (2019).

### 2.7 Biological Productivity Data

Net community productivity (mmol C · m^−3^ · day^−1^), or NCP, was determined by a surface layer budget for biologically active O_2_ based on mass spectrometer measurements of dissolved O_2_/Ar gas ratio and Winkler measurements of dissolved O_2_ concentrations in surface waters while on station. NCP is estimated following the procedure described by Emerson et al. (1991) and assumes steady‐state and negligible contribution by subsurface mixing. Air‐sea gas exchange rates needed to determine the air‐sea flux of biologically active O_2_ were estimated using satellite wind speeds at the location of each station (ASCAT data product at http://apdrc.soest.hawaii.edu/las/v6/) weighted over a 60‐day prior interval (Reuer et al., 2007) and the gas transfer rate dependence on wind speed of Wanninkhof et al. (2009). A stoichiometric O_2_/C ratio of 1.4 was assumed to express NCP in carbon units.

## 3 Results and Discussion

### 3.1 Diel Cycle of Cr (III)

**Figure 2:**
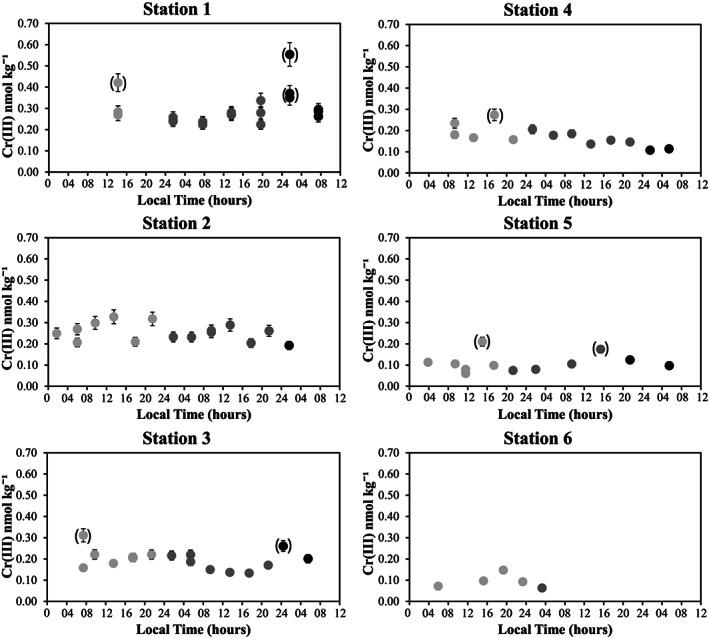
Diel Cr (III) measurements at 10‐m depth. Cr (III) concentrations from 10‐m depth are presented over 2 days (stations 1–5, 1 day for station 6) with local time of day. The light grey circles are day 1, the dark grey circles are day 2, and the black circles are day 3. Parentheses indicate identified outliers.

Samples for Cr redox speciation were collected every 4–8 hr for approximately 2 days at stations 1–5 and for 1 day at station 6. The starting time of the sampling varied depending on when a station was reached. However, complete cycles are available for every station, including at least 1 day for which data are continuously available from before dawn until the following morning. The data for diel cycle samples collected at a depth of 10 m are presented in Figure 2 and Table S3. While some variability is observed for [Cr (III)] across different points in time, no clear and consistent diel trends are observed over full diel cycles. A diel cycle for Cr redox species has been observed previously in an estuarine environment with anthropogenic influence (Kieber & Helz, 1992) and in lakes (Kaczynski & Kieber, 1993), with diel variability as high as 0.2 nmol/L and with both the presence of particulate material and sunlight identified as important factors in Cr reduction (Kaczynski & Kieber, 1993; Kieber & Helz, 1992). Higher sampling resolution and/or higher‐precision analyses may expose diel Cr redox cycling in the open ocean, or a diel cycle may not be apparent in such environments with lower concentrations of organics and lower anthropogenic influence. Regardless, our data demonstrate that diel variability of Cr (III) in open ocean environments is either minor or nonexistent.

Where full replicate samples are available (e.g., triplicates for each time point at station 1), they generally agree within ~10% (≤0.02 nmol/kg). However, there are also outliers, which may influence the ability to observe diel trends in stations where replicates were not collected for each time point. With no clear diel trend, a mean has been calculated for depths that were chosen for diel sampling (1–3 depths per station). This mean from diel replicates is the value used throughout our discussion, presented with an error of 1 *SD*. A difference was observed for [Cr (III)] at station 3, 75‐m depth between the first and second days of sampling (Tables S1 and S3). Other measured parameters (e.g., macronutrients and salinity; Table S1) are also different between days at this station, which is in the dynamic subarctic‐subtropical transition zone. The average of both days is presented in figures with [Cr (III)] data except for δ^53^Cr versus [Cr (III)] in Figures 4 and S2, where only the first day is shown for [Cr (III)] because δ^53^Cr is only available for the first diel cycle.

In looking at the entire [Cr (III)] dataset where diel replicates are available, ~10% of samples had significantly higher [Cr (III)]. These samples were determined to be statistical outliers by a modified Thompson τ test and were rejected from inclusion in the calculation of average [Cr (III)]. All diel cycle data are shown in Table S3, and outlying samples (shown in italic) are omitted from further discussion. Our discussion and interpretations are based primarily on the data for which means were calculated. While high values could be due to contamination, insufficient removal of the seawater supernatant is a more likely explanation given the low [Cr (III)]:[Cr] in our samples (roughly 5–10%). For [Cr (III)] = 5% of the total [Cr], the retention of approximated 0.5‐ml supernatant would contribute nearly as much Cr as the Cr (III) in the Mg (OH)_2_ pellet. Accidental loss of some precipitate during supernatant decanting could cause anomalously low values, though low statistical outliers were not observed, suggesting that this is not as much of a concern as insufficient supernatant decanting. From these results, it is advisable to rinse the precipitate with a Cr‐free solution (buffered to a high pH of 10–11) to ensure complete removal of seawater.

### 3.2 Distributions of [Cr] and [Cr (III)] in the Upper 200 m

**Figure 3:**
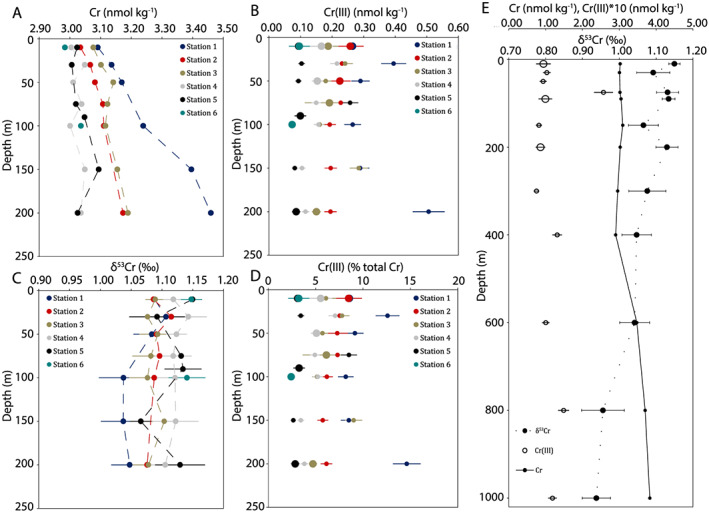
Depth profiles of [Cr], δ^53^Cr, and [Cr (III)]. Panel (a) shows [Cr], panel (b) shows δ^53^Cr, and panels (c) and (d) show absolute Cr (III) concentrations and percentages of total Cr in the topmost 200 m. Panel (e) shows profiles from station 5, where samples were collected to 1,000 m. Larger symbols in panels c–e denote samples for which diel [Cr (III)] replicates were measured (*n* = 5‐21) and are presented as means with 1 *SD*. Smaller symbols are individual measurements and are presented with 10% relative standard deviation.

Both [Cr] and [Cr (III)] decrease from higher to lower latitude stations (Figure 3). Subarctic stations and those at the subarctic‐subtropical transition zone generally display a slight [Cr] surface depletion relative to samples below 100 m, as observed previously in the subarctic North Pacific (Cranston, 1983). For stations in the subtropical North Pacific, depletions in [Cr] between the surface and 200 m are not observed, in agreement with published data (Moos & Boyle, 2019). At station 2 (Ocean Station PAPA/P26) our [Cr] and [Cr (III)] are similar to those previously reported at this site by Mugo and Orians (1993), though [Cr] at 10 m is lower (0.6 nmol/kg) than determined by Scheiderich et al. (2015).

Northern stations (1–4) show elevated [Cr (III)] above the winter mixed layer, with [Cr (III)] decreasing with depth, while [Cr (III)] remains stable with depth at the more southerly sites. In total, [Cr (III)] represents about 5–10% of [Cr] (Figure 3), in agreement with previous data from the North Pacific (Cranston, 1983; Cranston & Murray, 1978), with the rest of the dissolved Cr pool composed of Cr (VI) and possibly Cr (III) species, which are inert to particle adsorption (Cr (III)_NR_) (Nakayama et al., 1981). Chromium (III) data show the same trends whether expressed as absolute concentrations or as a percentage of [Cr] (Figure 3).

The surface [Cr (III)] maxima we observe (Figure 3), combined with the half‐life of Cr (III) in the ocean—estimated at roughly 0.5–2 months (Cranstron & Murray, 1980; Emerson et al., 1979; Pettine et al., 1991)—indicate that surface Cr (III) maxima are formed in situ. Previous studies have highlighted the importance of both light and particulate material in Cr reduction in surface fresh, estuarine, and ocean waters (Achterberg & van den Berg, 1997; Kaczynski & Kieber, 1993; Kieber & Helz, 1992). Indeed, known Cr reductants such as Fe (II) may be photochemically produced (e.g., Barbeau et al., 2001), and therefore, light could, at least in part, explain the observed surface [Cr (III)] maxima. However, differences in solar exposure over the range of our samples are low compared to the differences we observe in [Cr (III)] (>100%). Additionally, sunlight penetrates deeper in the subtopics, while [Cr (III)] decreases from the subarctic to the subtropics at all sampled depths. Therefore, the latitudinal gradient in [Cr (III)], both above the mixed layer and below the euphotic zone (Figure 3), argues against simple photochemical control on Cr reduction. Instead, these data can be explained by gradients in biological productivity.

**Figure 4:**
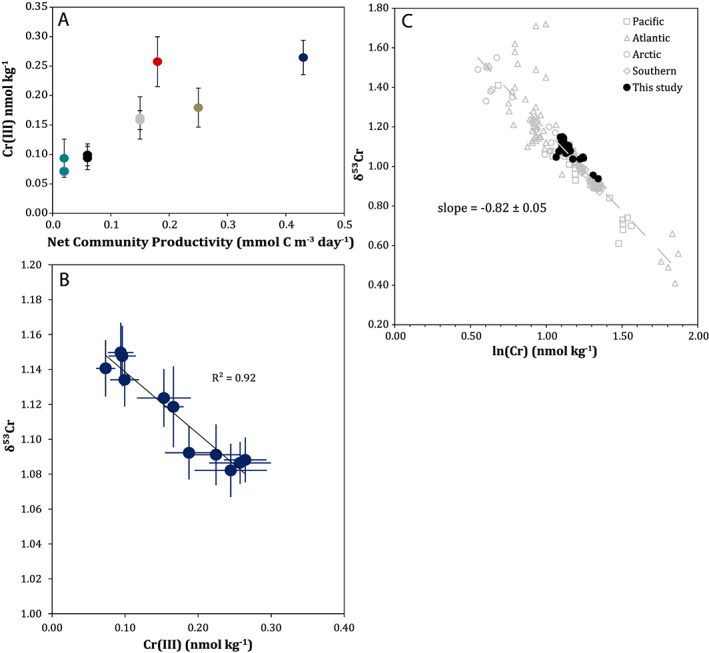
Relationship between biological productivity, [Cr (III)] and δ^53^Cr in the North Pacific and between δ^53^Cr and ln[Cr] in the global ocean. Panel (a) shows diel replicate [Cr (III)] from samples within the euphotic zone (above the 1% isolume, found at 40 to >100 m depending on the station; Table S4) and net community productivity. Panel (b) shows δ^53^Cr and [Cr (III)] above the winter mixed layer from stations and depths with [Cr (III)] diel replicates (see also Figure S2). The global δ^53^Cr‐ln[Cr] relationship, following Scheiderich et al. (2015), is shown in panel (c), including this study and literature data (Bonnand et al., 2013; Goring‐Harford et al., 2018; Moos & Boyle, 2019; Rickli et al., 2019; Scheiderich et al., 2015). Isolated surface samples and samples showing significant freshwater input have been omitted.

Within the euphotic zone, [Cr (III)] correlates with NCP (Figure 4a; *r* ^2^ = 0.76, *p* = 0.003), a measure of the new carbon fixed by photosynthesis minus autotrophic and heterotrophic respiration. In a steady state system where organic carbon is not accumulating in the surface ocean, NCP is equivalent to the biological export of organic carbon (Emerson, 2014). The correlation between [Cr (III)] and NCP, along with the similar residence time of Cr (III) (0.5–2 months) and the time over which NCP measurements integrate (1‐2 weeks), strongly supports a biological control of Cr reduction. This may result from a direct biological Cr reduction (Li et al., 2009) or an indirect biological influence (Kieber & Helz, 1992) through production of known Cr reductants (e.g., Fe (II): Maldonado et al., 2006). The release of Cr (III) during regeneration of exported biogenic particles, as has been suggested elsewhere (Achterberg & van den Berg, 1997), can explain elevated [Cr (III)] below the euphotic zone at stations with higher NCP.

**Figure 5:**
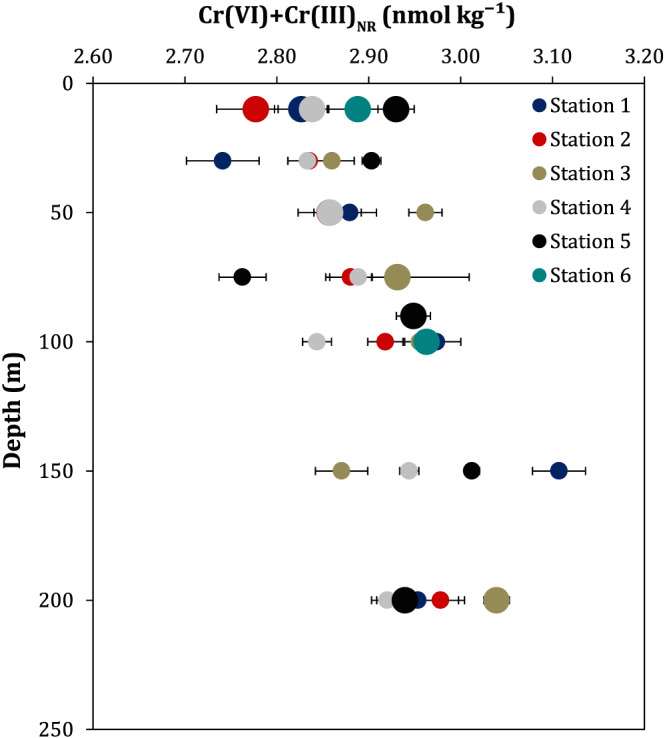
Depth profiles of [Cr (VI)] + [Cr (III)_NR_]. Large circles are data for which diel [Cr (III)] replicates are available (*n* =5 to 6 at station 6 and for samples from 200 m; *n* = 12 to 21 for other samples).

Concentrations of Cr (III) were subtracted from [Cr] to give concentrations of Cr (VI). This fraction would also include any Cr (III)_NR_, if such species are present. Profiles for [Cr (VI)] + [Cr (III)_NR_] are shown in Figure 5. A slight increase of [Cr (VI)] + [Cr (III)_NR_] with depth is apparent at higher latitude stations. However, while there is some variability between stations in [Cr (VI)] + [Cr (III)_NR_], a clear and consistent latitudinal [Cr (VI)] + [Cr (III)_NR_] trend at a given depth is not observed, in contrast to our [Cr (III)] and [Cr] data (Figure 3). Therefore, the consistent latitudinal gradient observed for [Cr] may be driven by [Cr (III)] gradients, and Cr (VI) (along with any Cr (III)_NR_) seem to behave more conservatively. Such semiconservative behavior of Cr (VI) has been highlighted previously (Sirinawin et al., 2000). Consequently, the production, adsorption, and export of the reactive Cr (III) species in areas of elevated biological productivity appear to exert control on [Cr] distributions.

### 3.3 Distributions of δ^53^Cr in the Upper 200 m, and δ^53^Cr, [Cr], and [Cr (III)] to 1,000 m

No significant variability of δ^53^Cr is found in depth profiles within the upper 200 m, except at station 1. Limited δ^53^Cr variability in the upper water column is in agreement with a previous depth profile in the North Pacific (Moos & Boyle, 2019). This likely reflects the only minor depletions of [Cr] in the upper water column, as the only station showing a δ^53^Cr difference outside of analytical uncertainty is station 1, with the largest observed [Cr] depletion. However, as with [Cr] and [Cr (III)], a latitudinal gradient is observed for δ^53^Cr, with isotopically lighter Cr found at higher latitudes (Figure 3). At 10‐m depth at station 2, δ^53^Cr is ~0.17‰ heavier and [Cr] is 0.6 nmol/kg lower than determined by Scheiderich et al. (2015). These differences may be explained by seasonal or interannual variability, as seasonal changes in [Cr] of a similar magnitude have been observed previously in the Atlantic (Connelly et al., 2006).

A profile to 1,000 m at station 5 (Figure 3) shows a nearly monotonic trend with increasing [Cr] and decreasing δ^53^Cr. These data better demonstrate [Cr] and δ^53^Cr variability with depth than the profiles to 200 m. Surface samples have lower [Cr] and are enriched in heavy δ^53^Cr, which supports earlier suggestions of a biological control involving the removal of isotopically light Cr from the surface ocean and regeneration of this isotopically light Cr at depth (Scheiderich et al., 2015). Minimal variability with depth is found for [Cr (III)], suggesting again that irradiance is not the most important direct factor in Cr (III) production. The persistence of very similar [Cr (III)] at depth at this low productivity site suggests that some factor may be acting to stabilize Cr (III) in deeper waters.

The absence of a perturbation to [Cr] and δ^53^Cr in the deepest samples (800–1,000 m), where oxygen concentrations are 10–20 μmol/kg, supports previous findings in the Pacific (Bruggmann, Klaebe, et al., 2019; Moos & Boyle, 2019; Wang et al., 2019) and the Atlantic (Goring‐Harford et al., 2018) that these O_2_ levels are not sufficiently low to drive large‐scale Cr removal. A slight depletion in [Cr] observed around 400 m may be related to North Pacific Intermediate Water (NPIW), which is identified at these depths by the salinity minimum around σ_θ_ = 26.6–27.0 (Talley, 1993). However, previous data for NPIW did not find a local [Cr] minimum (Moos & Boyle, 2019). The deepest samples at station 5 compare well with samples from the SAFe site (Moos & Boyle, 2019) and with samples from similar density surfaces from the Southern Ocean (e.g., Antarctic Intermediate Water [AAIW]; Rickli et al., 2019), though AAIW itself does not penetrate this far north in the Pacific (Fine et al., 2001; Qu & Lindstron, 2004).

Incorporating ours and other literature data (Goring‐Harford et al., 2018; Moos & Boyle, 2019; Rickli et al., 2019) to the initial open ocean δ^53^Cr‐ln[Cr] compilation (Scheiderich et al., 2015) yields a single linear trend with an isotope enrichment factor ε = −0.82 ± 0.05 (*r* ^2^ = 0.84; Figure 4c). This suggests biogeochemical Cr control by one process or by a limited range of processes with essentially equivalent stable isotope fractionation (Scheiderich et al., 2015). And, as discussed above, available data suggest that OMZ Cr removal in the modern ocean may not be the dominant process governing δ^53^Cr. We therefore assess the potential of biological cycling as a mechanism to explain our data.

### 3.4 Deficits of Cr in Mixed Layer and Inferred Biological Cr Export

The Cr deficit in the upper 100 m can be quantified and related to biological export. To determine the magnitude of the deficit, the Cr inventory in the upper 100 m (the approximate depth of the winter mixed layer; Freeland et al., 1997; Whitney & Freeland, 1999) is calculated by trapezoidal integration, converted to a representative averaged [Cr] in the upper 100 m, and subtracted from the average [Cr] from 150 to 200 m. This deficit of Cr, in units of nmol/kg, is significant for stations 1–3 considering the analytical uncertainty in [Cr]. Concentration deficits were transformed to areal deficits (in μmol Cr/m^2^) within the mixed layer at the time of sampling.

We then determined if these deficits can be explained by biological export from the surface ocean by multiplying annual net community productivity (as mol C · m^−2^ · year^−1^, determined within the average mixed layer over two diel cycles on station) by approximate phytoplankton Cr:C ratios as observed in natural oceanic phytoplankton assemblages (2.7 μmol Cr mol/C; Martin & Knauer, 1973, based on a median Cr content of 3.9 μg/g dry weight and assuming 33 wt. % C, as applied in Martin et al., 1989) and in Cr:C observed in sediment traps at 3,200‐m water depth (0.555 μmol Cr mol/C; Connelly et al., 2006; Table 1). The calculation is approximate, given uncertainty in extrapolating seasonal NCP to annual productivity data (e.g., Ayers & Lozier, 2010; Emerson, 2014; Juranek et al., 2012) as well as limited data for phytoplankton Cr:C. However, our inferred exported Cr range encompasses the observed Cr deficit at all stations (Table 1), excepting one station where the “observed depletion” is in fact not significant within analytical uncertainty. Therefore, biological export of Cr at observed Cr:C ratios of marine phytoplankton and exported particles can explain the observed surface drawdown of [Cr], suggesting that Cr cycling in the surface ocean is linked to biological activity and the C cycle.

**Table 1:** Comparison of Surface Ocean Cr Deficits and Inferred Exported Cr as Well as Global Cr Export

Station	Latitude	[Cr] deficit	Mixed layer	Cr deficit (ML)	ANCP	Phytoplankton Cr:C^a^	Inferred exported Cr
	°N	nmol/kg	m	μmol/m^2^	mol C · m^−2^ · year^−1^	μmol Cr:mol C	μmol · m^−2^ · year^−1^
1	55	0.27	19.2	4.1	3.02	0.56–2.7	2–8
2	50	0.21	33.8	3.2	2.22	0.56–2.7	1–6
3	46	0.06	41.5	2.5	3.79	0.56–2.7	2–10
4	42	0.01*	28.7	0.3	1.57	0.56–2.7	0.9–4
5	34	0.04*	26.9	1.2	0.59	0.56–2.7	0.3–2
6	24	NA	39.7	NA	0.29	0.56–2.7	0.2–0.8
		Biological carbon export			Cr export	Cr sources^b^	
		GT C/year			mol Cr/year	mol Cr/year	
Global ocean—surface^c^		5–8			2.3–13 × 10^8^	0.8–5.6 × 10^8^	
Global ocean—2,000 m^d^		0.4			0.2–1 × 10^8^		

*Note*. ML is the mixed layer while on station. ANCP is annual net community productivity. NA is no data below 100 m to calculate a deficit. The asterisk indicates that deficit is not significant when considering 1 relative standard deviation error for concentration measurements (0.82%), implying that there is no strong evidence for surficial Cr depletion at these sites.

a Cr:C from Martin and Knauer (1973; surface Pacific phytoplankton) and Connelly et al. (2006); sediment traps at 3,200 m).

b Bonnand et al. (2013).

c Henson et al. (2011), Laufkötter et al. (2013), and Laws et al. (2000) as presented in Honjo et al. (2008).

Honjo et al. (2008).

Semeniuk et al. (2016) presented a similar calculation. Our calculation differs in two main ways: (1) Our [Cr] deficits are calculated comparing water above the winter mixed layer and the immediately underlying waters rather than comparing the mixed layer to waters at ≥500 m, and therefore our calculated Cr deficit is smaller; and (2) we use phytoplankton Cr data from the open North Pacific (Martin & Knauer, 1973) and exported particles in the open North Atlantic (Connelly et al., 2006), rather than data from North Atlantic marginal seas (Dauby et al., 1994), which show a much higher Cr:C (~20 μmol Cr mol/C) and may not be representative of the open ocean.

Extrapolating this calculation to the global ocean allows for a comparison of biological Cr export from the oceans to oceanic inputs. Combining the Cr:C ratios mentioned above with estimations of the strength of the biological pump, or equivalently the total global biological carbon export from the surface ocean, of 5–8 GT C/year (from Henson et al., 2011—5 GT C/year; Laufkötter et al., 2013—8 GT C/year; and Laws et al., 2000, as presented in Honjo et al., 2008—5.7 GT C/year), and the carbon flux observed at 2,000 m (Honjo et al., 2008—0.43 GT C/year) yields a surface export of 2.3–13 × 10^8^ mol Cr per year, and an export of 0.2–1 × 10^8^ mol Cr per year at 2,000 m (Table 1). These fluxes are approximately the same as the total riverine flux of Cr to the ocean (0.5–5.6 × 10^8^ mol Cr/year; Bonnand et al., 2013), which accounts for 76–96% of the total Cr sources to the ocean (Bonnand et al., 2013). This close agreement between the estimated biologically driven Cr export and riverine input fluxes suggests that biologically driven Cr flux is an important process, if not the dominant process, in the oceanic Cr cycle. Jeandel and Minster (1987) estimated biological Cr flux from the surface ocean on the order of ~10 × 10^8^ mol Cr/year. The similarity in our value and theirs is noteworthy, considering the advances in data available to constrain components of the Cr cycle and that different approaches were used to reach these values.

### 3.5 Chromium Reduction and Scavenging of Cr (III) as a Putative Control of [Cr] and δ^53^Cr in the Surface Ocean

The first [Cr] depth profiles from the ocean suggested a biological control (Campbell & Yeats, 1981; Cranston, 1983; Jeandel & Minster, 1984), and a connection has previously been observed between [Cr (III)] and productivity in surface waters as well as [Cr] and POC in exported particles (Connelly et al., 2006; note that these authors also concluded that Cr export had a minor impact on the local Cr cycle overall). Additionally, initial interpretations of [Cr] and δ^53^Cr data invoked biological control on δ^53^Cr‐ln[Cr] systematics (Scheiderich et al., 2015). However, this was based on isolated surface and subsurface measurements, and subsequent full‐depth profiles have highlighted a lack of strong support for biological export and regeneration at depth as a major factor in global δ^53^Cr and [Cr] distributions (Goring‐Harford et al., 2018; Moos & Boyle, 2019; Rickli et al., 2019). Therefore, while the potential role of biological productivity in driving [Cr] and δ^53^Cr is not entirely new, data available at present have not been able to clearly establish biological cycling as a prominent control on [Cr] and δ^53^Cr. We present the first assessment of biological cycling in open ocean environments involving Cr redox speciation, [Cr], δ^53^Cr, and direct measurements of biological production.

We observe a linear relationship between [Cr (III)] and total dissolved δ^53^Cr above the winter mixed layer (Figure 4b). This suggests that the removal of isotopically light Cr (III) can explain the transition we observe of light to heavy δ^53^Cr from the subarctic to the subtropical North Pacific (Figure 3), where [Cr] decreases without a consistent change in Cr (VI)+Cr (III)_NR_ (Figure 5). The linear relationship between Cr (III) and δ^53^Cr above the winter mixed layer across our samples can be used to provide a first‐order estimate of the isotopic offset for Cr (III) relative to the dissolved pool in the upper ocean. While such a linear relationship is unlikely to hold true over a wide range of [Cr (III)] and δ^53^Cr, and when [Cr (VI)+Cr (III)_NR_] varies significantly, we assume that our samples reflect a quasi‐linear trend due the restricted range of [Cr (III)], proximity to [Cr (III)] = 0, the absence of a clear and uniform trend in [Cr (VI) + Cr (III)_NR_], and the strength of a simple linear fit (*r* ^2^ = 0.92; see the supporting information for further discussion).

With the assumption of constant [Cr (VI) + Cr (III)_NR_] (2.9 nmol/kg) and constant δCr53CrVI&CrIIINR over the range of our data, and a linear trend relating total dissolved δ^53^Cr and [Cr (III)], δ^53^Cr for Cr (III) removal and addition can be calculated based on the following mass balance equations:

(2) $$\left[\mathrm{Cr}\right]=\left(\left[\mathrm{Cr}\left(\mathrm{VI}\right)+\mathrm{Cr}{\left(\mathrm{III}\right)}_{\mathrm{NR}}\right]\right)+\left[\mathrm{Cr}\left(\mathrm{III}\right)\right]$$

(3) δCr53×Cr=δCr53CrVI+CrIIINR×CrVI+CrIIINR+δCr53CrIII×CrIII

From the intercept of the δ^53^Cr and [Cr (III)] trend, we calculate a δ^53^Cr for Cr (VI)+Cr (III)_NR_ of 1.178‰ ± 0.015 (95% confidence interval). This yields a ∆^53^Cr = −1.08‰ ± 0.25 for the δ^53^Cr of Cr (III) addition and removal compared to total dissolved δ^53^Cr. This value overlaps with the global enrichment factor for Cr addition and removal determined from the δ^53^Cr‐ln[Cr] trend of ε = −0.82 ± 0.05 (95% confidence interval, including reported uncertainties on δ^53^Cr and assuming 1% for [Cr] measurements; data from Bonnand et al., 2013; Scheiderich et al., 2015; Goring‐Harford et al., 2018; Moos & Boyle, 2019; Rickli et al., 2019; and this study). The inferred ∆^53^Cr also matches that observed for Cr reduction by Fe (II) in laboratory settings under continuous Fe (II) supply (Døssing et al., 2011), which reflects Fe (II) conditions relevant for the surface ocean, and supports a potential role for Fe (II) in Cr reduction in the surface ocean.

We note that this calculation assesses the Cr (III) mass balance, or the impact of adding or removing Cr (III) from the bulk dissolved pool (i.e., moving left or right in Figure 4b), and therefore combines any isotopic offset between δCr53CrVI&CrIIINR and δCr53CrIII with any fractionation associated with Cr (III) removal (i.e., biological uptake and scavenging). Therefore, this calculation reflects the sum of these processes, and not necessarily the difference between δCr53CrVI&CrIIINR and δCr53CrIII. Additionally, given the fractionations associated with Cr reduction, we expect that the presence of significant Cr (III)_NR_, and any fractionations associated with any organic complexation rendering Cr (III) nonreactive, may also result in differences between what is reflected in our calculation and differences between δCr53CrVI, δCr53CrIIINR, and δCr53CrIII.

This influences comparability between our calculated value and measurement of redox‐specific δ^53^Cr. Indeed, a smaller ∆^53^Cr is observed in recently available redox‐specific δ^53^Cr data (∆^53^Cr = −0.42‰ ± 0.15, *n* = 5 samples; Wang et al., 2019), though the dataset for such comparisons is still small, and these available redox‐specific Cr data deviate from previous [Cr (III)] and [Cr (VI)] measurements in the region (Murray et al., 1983; Rue et al., 1997) and from δ^53^Cr and [Cr] elsewhere in the eastern North Pacific (Scheiderich et al., 2015; Moos & Boyle, 2019; this study). Further redox‐specific δ^53^Cr data may better elucidate any differences that may exist between field data and our calculation, and mechanisms responsible for this. Nevertheless, the net Cr removal and addition processes, including any fractionations in component steps of the overall process, are the important determinants for understanding the observed δ^53^Cr‐ln[Cr] trend, which reflects the isotopic impact of Cr addition or removal (i.e., moving left or right in Figure 4c).

Taken comprehensively, (a) the correlation between [Cr (III)] and biological productivity, (b) the agreement between observed [Cr] deficits and expected biological Cr export, (c) the relationship between [Cr (III)] and δ^53^Cr, and (d) the similarity between the calculated ∆^53^Cr for Cr (III) removal and the fractionation factor for Cr addition and removal in the global δ^53^Cr‐ln[Cr] dataset provide evidence for a biological control of the oceanic Cr biogeochemical cycle involving a link between biological productivity, Cr reduction and export, and stable isotope fractionation. The imprint of biological processes on water masses can be further modified by processes such as regeneration from biogenic particles, water mass circulation and mixing (e.g., Rickli et al., 2019; Scheiderich et al., 2015), and in coastal environments and restricted basins, localized impacts including riverine sources (e.g., Paulukat et al., 2016), acting to shape the global δ^53^Cr‐ln[Cr] distribution (see also section 3.3). The impacts of these processes should become clearer as the global δ^53^Cr dataset grows. Biological control on Cr biogeochemical cycling in the modern open ocean reveals a potential role for Cr isotopes as tracers of biological productivity in both the modern ocean and in the past.

## 4 Paleoceanographic Potential and Implications

A biological control on global δ^53^Cr provides two important implications for paleoceanographic applications of δ^53^Cr: (i) biological modification of oceanic δ^53^Cr complicates interpretations when using δ^53^Cr records to reconstruct oxygen availability and (ii) δ^53^Cr records may have utility as a proxy for biological productivity. In reference to (i), we stress that with significant redox gradients during the oxygenation of the Earth and when widespread anoxia was present, oxygen availability is likely the proximate control on δ^53^Cr. However, in conditions such as those found in the modern ocean and throughout the Late Quaternary, without widespread anoxia (Jaccard & Galbraith, 2012), δ^53^Cr records may not primarily record O_2_ availability. Because δ^53^Cr records in marine sediments from the late Quaternary are at times interpreted through an O_2_‐controlled framework, even when δ^53^Cr records correlate with other proxies known to be influenced by biological activity (δ^15^N) and do not correlate with records of local O_2_ availability (e.g., Gueguen et al., 2016), the role of biological controls on δ^53^Cr may at times be underestimated in reconstructions using sedimentary δ^53^Cr records.

Below we assess the potential for δ^53^Cr to act as a productivity proxy as well as limitations due to current gaps in the understanding of Cr and δ^53^Cr cycling. This discussion is structured around the following criteria, which must be met for δ^53^Cr to serve as a useful productivity proxy: (1) biological export of Cr from the surface ocean is correlated to export of organic carbon, (2) a consistent trend is observed between Cr removal and δ^53^Cr, and (3) δ^53^Cr is preserved in sediments. We note that restricted basins may reflect localized inputs and deviate from expected open ocean ln[Cr]‐δ^53^Cr trends (Paulukat et al., 2016), and therefore, any paleoproductivity proxy utility that δ^53^Cr may have would be restricted to environments with Cr behavior analogous to the modern open ocean.

**Figure 6:**
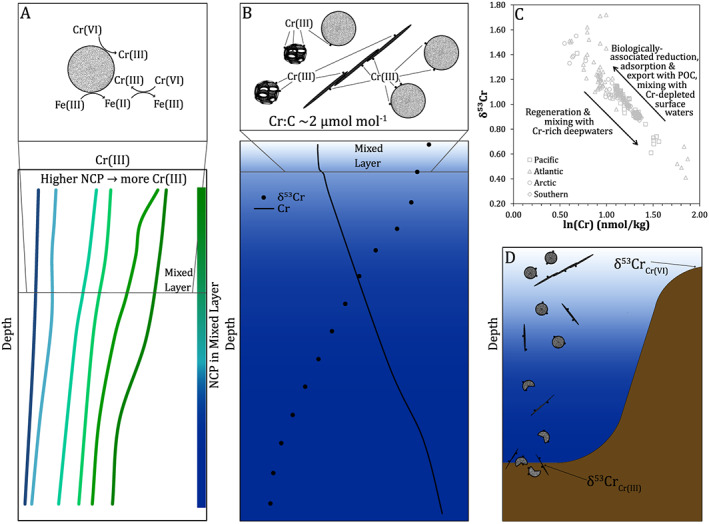
Model for biological control on the Cr cycle and potential paleoceanographic applications. (a) Surface waters with higher NCP have higher [Cr (III)] (lower panel). This can be explained by biological reduction of Cr, either directly (Li et al., 2009) or indirectly through production of known Cr reductants (e.g., Fe (II), Maldonado et al., 2006). (b) Surface waters show [Cr] deficits and heavy δ^53^Cr relative to deeper waters (lower panel), likely due to adsorption and/or biological uptake of Cr (III) in surface waters (Semeniuk et al., 2016; upper panel). While surface Cr drawdown is less than for other biologically cycled elements, Cr export from the surface ocean associated with organic carbon, with a Cr:C ratio observed for natural open ocean phytoplankton assemblages (~2 μmol Cr/mol C; Martin & Knauer, 1973) explains observed surface ocean Cr deficits. Biologically mediated export of Cr also approximately balances known Cr sources, and therefore is likely a major Cr sink, if not the major sink, in the modern ocean. (c)The global δ^53^Cr‐ln[Cr] correlation implies an isotope enrichment factor that agrees with what we calculate in surface waters associated with the removal of Cr (III). Our data support that the observed δ^53^Cr‐ln[Cr] relationship can be controlled by Cr reduction and export from surface waters, as well regeneration from biological particle flux and water mass mixing. (d) If biological activity controls the distribution of [Cr] and δ^53^Cr, then records of δ^53^Cr may serve as a paleoceanographic proxy for biological productivity. This can be applied with (1) δ^53^Cr in shallow water carbonates, which record surface δ^53^Cr when corrected for any vital effects, or (2) δ^53^Cr delivered to sediments along with organic carbon flux, reflecting isotopically light Cr (III) scavenged onto particles.

(1) Data from sediment traps (Connelly et al., 2006; Francois, 1988) and marine sediments (Francois, 1988) indicate that export of Cr is correlated with export of organic carbon. Laboratory incubations with marine algal isolates demonstrate that Cr (III) is adsorbed onto phytoplankton cells (Semeniuk et al., 2016), providing a mechanism for Cr‐POC coupling. Building on these data, we present evidence that biological activity in the surface ocean increases [Cr (III)] (section 3.2), as has also been shown in the Atlantic Ocean (Connelly et al., 2006) and that observed Cr deficits agree with those expected from a proxy for biological carbon export (section 3.4; Table 1 and Figures 6a and 6b). Furthermore, biological Cr export expected from global export production is of a similar magnitude to the total global Cr sources (Table 1), a result also found by Jeandel and Minster (1987) using a different approach. Therefore, biological Cr export linked to carbon export explains our [Cr] observations and may be the main global Cr sink.

Criterion (2) is analogous to the application of nitrogen stable isotopes (δ^15^N) as a paleoproxy for biological productivity (e.g., Altabet & Francois, 1994). In this case, progressive removal of light isotopes by biological activity in the surface ocean generates an increasingly fractionated isotope composition. Therefore, it is not only the present biological activity which drives the isotope composition of the surface ocean but the cumulative biological export that a water parcel has seen since reaching the euphotic zone. For δ^53^Cr to work accordingly, the removal of Cr by biological export must result in a consistent and uniform increase in δ^53^Cr in surface waters. The strong linear correlation in global δ^53^Cr‐ln[Cr], spanning four of the five ocean basins (Figure 4c), gives evidence of a single consistent δ^53^Cr fractionation trend (Scheiderich et al., 2015). Indeed, Scheiderich et al. (2015) postulated that biological export was one of the main reasons for the global δ^53^Cr‐ln[Cr] trend, though data were limited to test this at the time and clear support has not been apparent in subsequent open ocean δ^53^Cr depth profiles (Goring‐Harford et al., 2018; Rickli et al., 2019). The NCP‐[Cr (III)] and δ^53^Cr‐[Cr (III)] relationships we observe (Figure 4 and sections 3.2 and 3.5), combined with the similar fractionations in global δ^53^Cr‐ln[Cr] data and that we infer for Cr (III) removal, gives support that the biological export of Cr from the surface ocean follows this fractionation (Figure 6c), though the limited δ^53^Cr range across modern ocean surface waters may restrict potential proxy applicability.

With respect to criterion (3) laboratory experiments demonstrate that Cr exported with biogenic particles is predominantly Cr (III) (Semeniuk et al., 2016). Previous data showing correlations between biological activity and Cr (III) in surface waters (Connelly et al., 2006) and our field data, showing a strong correlation between NCP and [Cr (III)] as well surface [Cr] deficits, support these laboratory findings. Therefore, the isotopic signature of Cr exported with organic carbon would reflect isotopically light Cr (III), a record which may be preserved in marine sediments. Considerable work has been conducted to assess if δ^53^Cr in carbonates, which incorporate Cr (VI) (Tang et al., 2007), reflects ambient seawater δ^53^Cr (Bonnand et al., 2013; Bruggmann et al., 2019; Farkaš et al., 2018; Frei et al., 2018; Holmden et al., 2016; Pereira et al., 2016; Remmelzwaal et al., 2019; Rodler et al., 2015; Wang et al., 2017). Similar work is needed to determine how δ^53^Cr records in oxic marine sediments relate to δ^53^Cr in the overlying water column and how faithfully these records are preserved (Figure 6d). This may include assessing the differences between redox‐specific δ^53^Cr of Cr (III) and Cr (VI), investigations of any fractionation in the scavenging of Cr (III), and the degree to which exported δ^53^Cr associated with POC flux is preserved during respiration, partial oxidation and diagenesis. Such work will also have to assess the precision of δ^53^Cr records and to what extent these are able to resolve different surface δ^53^Cr signals.

## 5 Conclusions

Surface maxima are found for [Cr (III)] in higher productivity stations, while stations with lower NCP do not show [Cr (III)] maxima in the sunlit surface waters. Elevated Cr (III) is also seen below the euphotic zone at high productivity stations relative to low productivity stations. Given these trends above and below the euphotic zone, it does not appear that irradiance is a primary driver of Cr reduction. Rather, our data indicate that Cr is reduced in the surface ocean, either directly or indirectly, by biological activity. Subsurface [Cr (III)] trends can be explained by regeneration of Cr (III) from exported biogenic particles. In contrast to data from freshwater and estuarine systems (Kieber & Helz, 1992; Kaczynski & Kieber, 1993), no diel Cr (III) variability is seen in the open ocean. Dissolved [Cr] decreases from high latitude to low latitude, and higher latitude stations show a Cr deficit above the winter mixed layer. This can be explained by biological export at Cr:C ratios reported in marine phytoplankton (Martin & Knauer, 1973) and sinking particles (Connelly et al., 2006). Stations with lower NCP do not show Cr deficits outside of analytical uncertainty, in agreement with expected export based on measured NCP and reported Cr:C. This gives strong support to biological productivity controlling Cr (III) fractions in and Cr export from the surface ocean. Based on total annual global carbon export, we calculate a global annual Cr export of 2.3–13 × 10^8^ mol Cr/year from the surface ocean and 0.2–1 × 10^8^ mol Cr/year at 2,000‐m depth, of similar magnitude to global Cr sources (0.8–5.6 × 10^8^ mol Cr/year; Bonnand et al., 2013). Therefore, biological export is likely one of primary Cr sinks, if not the primary sink, in the global ocean.

We find almost no variability with depth within the upper 200 m for δ^53^Cr considering analytical uncertainty. The latitudinal trend in δ^53^Cr is, however, significant. A profile to 1,000 m in the subtropical North Pacific shows monotonically decreasing δ^53^Cr and increasing [Cr], in support of a biological control on δ^53^Cr. Dissolved [Cr] and δ^53^Cr data correspond well with the linear global δ^53^Cr‐ln[Cr] relationship (Scheiderich et al., 2015), with an ε = −0.82 ± 0.05. No influence on [Cr] or δ^53^Cr is seen in low oxygen waters in our data, in support of other recent studies indicating that oceanic OMZs may not exert large‐scale control on oceanic [Cr] and δ^53^Cr distributions (Bruggmann, Klaebe, et al., 2019; Goring‐Harford et al., 2018; Moos & Boyle, 2019; Wang et al., 2019). A strong linear relationship is observed between δ^53^Cr and [Cr (III)], which suggests that removal of Cr (III) results in increasing δ^53^Cr. From this we calculate an isotope enrichment factor (∆^53^Cr) = −1.08‰ ± 0.25 for removal of Cr (III). The agreement between our ∆^53^Cr and the observed trend in the global δ^53^Cr‐ln[Cr] relationship, combined with the evidence we find of a biological control on Cr redox speciation and concentrations, supports a biological control on δ^53^Cr in the global ocean. This presents a new potential for δ^53^Cr records in marine sediments to serve as a proxy for biological productivity and complicates interpretations of marine‐origin δ^53^Cr records to infer redox conditions and oxygen availability under oceanic conditions without widespread anoxia.

## Author Contributions

DJJ and SLJ planned the study. PDQ and AEW planned and coordinated shipboard sampling operations, and DJJ planned Cr sampling. DJJ collected Cr samples, PDQ collected and analyzed NCP samples, and AEW collected and analyzed NO_3_^−^ samples. DJJ and JR analyzed the Cr samples. PN contributed seawater Cr standard measurements to the compilation used to assess external precision. DJJ, SLJ, and JR interpreted the Cr data. All authors contributed to the manuscript.

## Supporting information

Supporting Information S1
