# Supplementary material for: Biological Control of Chromium Redox and Stable Isotope Composition in the Surface Ocean
Source: Global Biogeochem Cycles. 2020 Jan 16;34(1):e2019GB006397. doi: 10.1029/2019GB006397 (PMC7375040; doi:10.1029/2019GB006397)
Supplement: Supplementary file 1 — Supporting Information S1 [file GBC-34-e2019GB006397-s001.docx]

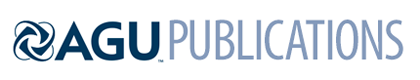


*Global Biogeochemical Cycles*

Supporting Information for

Biological control of chromium redox and stable isotope composition in the surface ocean

David J. Janssen^1*^, Jörg Rickli^1,2^, Paul D. Quay^3^, Angelicque E. White^4^, Philipp Nasemann^1^, Samuel L. Jaccard^1^

^1^University of Bern, Institute of Geological Sciences & Oeschger Center for Climate Change Research, Baltzerstrasse 1-3, 3012, Bern Switzerland

^2^Institute of Geochemistry and Petrology, ETH Zürich, Clausiusstrasse 25, 8092, Zürich Switzerland

^3^University of Washington, School of Oceanography, 1503 NE Boat Street, Seattle, WA 98195, USA

^4^University of Hawai’i at Manoa, School of Ocean & Earth Science and Technology, 1950 East West Road

Honolulu, HI 96822, USA

^*^Corresponding author: david.janssen@geo.unibe.ch; janssen.davej@gmail.com

**Contents of this file**

Text S1 to S2

Figures S1 to S2

Tables S1 to S4

**Introduction**

This supplemental material includes additional tables and figures, as well as specific calculations details which are summarized in shorter form in the main text. Full data tables are available from Zenodo at doi: 10.5281/zenodo.3560082 as well as below in the supplemental material.

**S.1 External Reproducibility of [Cr] and δ^53^Cr**

Assessments of external reproducibility are presented below. This is accomplished in two ways: (i) replicate measurements of standard solutions at the target Cr concentration range for sample analysis, and (ii) replicate measurements of samples. In the former case, this presents a large sample size where one sample/standard has been repeatedly measured; however it is also an idealized situation where the sample matrix is absolutely pure (only Cr from stock solutions and the pure acid used for dissolving samples), and does not account for any variability in sample processing/matrix and how this may influence analyses. Therefore, this approach is likely an underestimation of the actual analytical variability expected for samples. The external reproducibility from NIST SRM 979 standards analyzed during this study is shown in Figure S.1 and is 0.021‰ (n = 77, 2 SD).

Replicate measurements of samples provide a more realistic assessment of reproducibility. However, there are no samples for which a sufficiently large number of aliquots have been processed through chemistry and analyzed, therefore presenting statistical limitations to assess external reproducibility. This is compensated for by combining the data from a sufficiently large pool of samples which have each been replicated a smaller number of times, following Kenney & Keeping (1951). This approach assesses the variability from the mean for each set of replicates and combines this information to determine the overall reproducibility. The full set of [Cr] and δ^53^Cr replicates used for this determination is shown in Table S.2, including replicates and seawater intercalibration standards presented in Rickli et al. (2019, data given in italic). For δ^53^Cr we calculate an external reproducibility of 0.0328‰, (2 SD) based on 49 measurements of 17 samples/seawater standards. For [Cr], we calculate an external reproducibility of 0.82% (1 RSD) based on 49 measurements of 17 samples.

**S.2 δ^53^Cr and [Cr(III)]**

A linear relationship is observed between Cr(III) and δ^53^Cr, which suggests that processes that add or remove Cr(III) influence δ^53^Cr of the total dissolved Cr pool and that variable proportions of Cr(III) are sufficient to explain observations in total dissolved δ^53^Cr. We caution that this specific relationship is unlikely to hold in other regions and under different ranges of [Cr(III)] and [Cr(VI) + Cr(III)_NR_], though different correlations between [Cr(III)] and δ^53^Cr may be found. This section describes the quantitative treatment of that relationship. All data above the winter mixed layer show a similar trend as seen in Figure 4 (Figure S.2). However, as described in section 3.1 (*Diel Cycle of Cr(III)*), greater confidence is given to stations and depths for which several Cr(III) samples were collected (either as actual replicates, or in the time series sampling) due to uncertainties in individual [Cr(III)] measurements. Therefore, only the depths with replicate Cr(III) determinations are used for the quantitative treatment of this relationship.

A simple linear regression of the data demonstrate a robust relationship (r^2^ = 0.92, Figure 4). Taking into account the uncertainties in both δ^53^Cr and [Cr(III)] in the individual measurements, this relationship yields the trendline δ^53^Cr = (-0.373‰ ± 0.086 (nmol kg^-1^)^-1^) $\times$ [Cr(III)] + (1.178‰ ± 0.015) at the 95% confidence interval. The R package Dreming was employed to determine the trendline, which is used for quantitative treatment of the data, including calculations of Δ^53^Cr for Cr(III) removal. The quantitative treatment relies on the following assumptions, observations, and equations:

1. For this calculation, a constant value of [Cr(VI) + Cr(III)_NR_] of 2.9 nmol/kg is assumed. The concentration ranges between stations and within depth profiles for [Cr(VI) + Cr(III)_NR_] are smaller than observed for [Cr] and [Cr(III)]. Furthermore, no consistent trend across stations is observed, unlike those found for both [Cr] and [Cr(III)]. Therefore, [Cr(VI) + Cr(III)_NR_] appears to behave more conservatively than [Cr] and [Cr(III)], with differences in [Cr] driven by differences in [Cr(III)], and the assumption of stable [Cr(VI) + Cr(III)_NR_] is supported by our data.
2. As a corollary to the relationship between [Cr] and δ^53^Cr from the global ocean (Scheiderich et al., 2015), where a consistent relationship is observed between [Cr] and δ^53^Cr, ${\delta{}^{53}\mathrm{Cr}}_{\mathrm{Cr}\left( \mathrm{VI} \right) \& {\mathrm{Cr}\left( \mathrm{III} \right)}_{\mathrm{NR}}}$ is assumed to be constant when [Cr(VI) + Cr(III)_NR_] is constant. ${\delta{}^{53}\mathrm{Cr}}_{\mathrm{Cr}\left( \mathrm{VI} \right) \& {\mathrm{Cr}\left( \mathrm{III} \right)}_{\mathrm{NR}}}$ is determined from the intercept of the trendline for δ^53^Cr versus [Cr(III)] (Equation 1).
3. Processes that add or remove Cr(III), and therefore shift total dissolved δ^53^Cr to higher or lower values (i.e. moving left and right in Figures 4 and S.2), may combine isotopic offsets between the reactive ${\delta{}^{53}\mathrm{Cr}}_{Cr(III)}$ pool and the more conservative ${\delta{}^{53}\mathrm{Cr}}_{\mathrm{Cr}\left( \mathrm{VI} \right) \& {\mathrm{Cr}\left( \mathrm{III} \right)}_{\mathrm{NR}}}$ pool with isotope fractionations within the addition or removal process itself. Therefore calculations based on the observed relationship combine the two potential fractionation processes, and no attempt is made to differentiate between these two with the information currently available.
4. For a given condition of δ^53^Cr and [Cr(III)] based on the observed trendline (Equation 1), the isotopic offset (Δ^53^Cr) between ${\delta{}^{53}\mathrm{Cr}}_{Cr(III)}$ and total dissolved δ^53^Cr can be calculated by mass balance (Equations 2 and 3)

δ^53^Cr = (-0.373‰ ± 0.086 (nmol kg^-1^)^-1^) $\times$ [Cr(III)] + (1.178‰ ± 0.015) (Equation 1)

$\left[ \mathrm{Cr} \right]= \left( \left[ \mathrm{Cr}\left( \mathrm{VI} \right)+ {\mathrm{Cr}\left( \mathrm{III} \right)}_{\mathrm{NR}} \right] \right)+[Cr\left( \mathrm{III} \right)]$ (Equation 2)

$\delta{}^{53}\mathrm{Cr}\times\left[ \mathrm{Cr} \right]={\delta{}^{53}\mathrm{Cr}}_{\mathrm{Cr}\left( \mathrm{VI} \right)+{\mathrm{Cr}\left( \mathrm{III} \right)}_{\mathrm{NR}}}\times\left( \left[ \mathrm{Cr}\left( \mathrm{VI} \right)+{\mathrm{Cr}\left( \mathrm{III} \right)}_{\mathrm{NR}} \right] \right)+{\delta{}^{53}\mathrm{Cr}}_{\mathrm{Cr}\left( \mathrm{III} \right)}\times[Cr\left( \mathrm{III} \right)]$ (Equation 3)

*
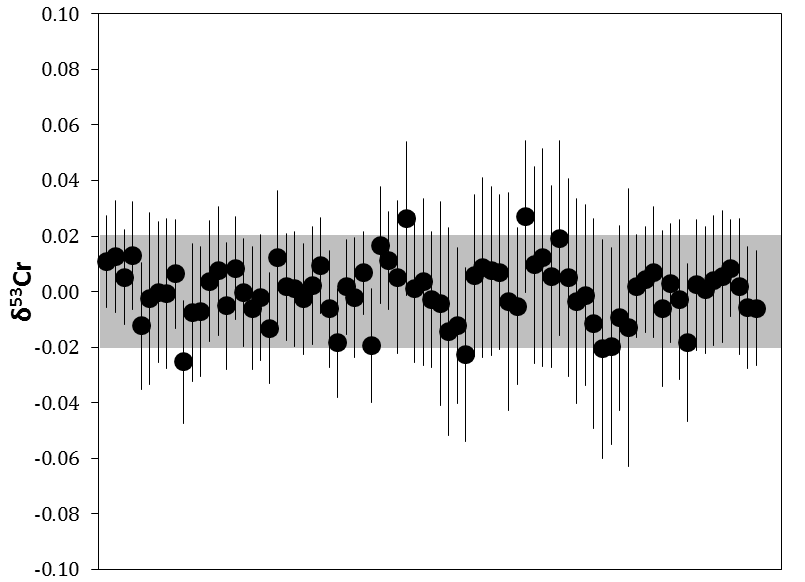
*

**Figure S.1: Spiked NIST SRM 979 standards**. All NIST SRM 979 standards analyzed for this study, corrected to δ^53^Cr = 0.00‰ by the daily average value, are shown with their internal error (2 SEM). The grey box shows the mean ± 2 SD, 0.021‰, for all 77 measurements.


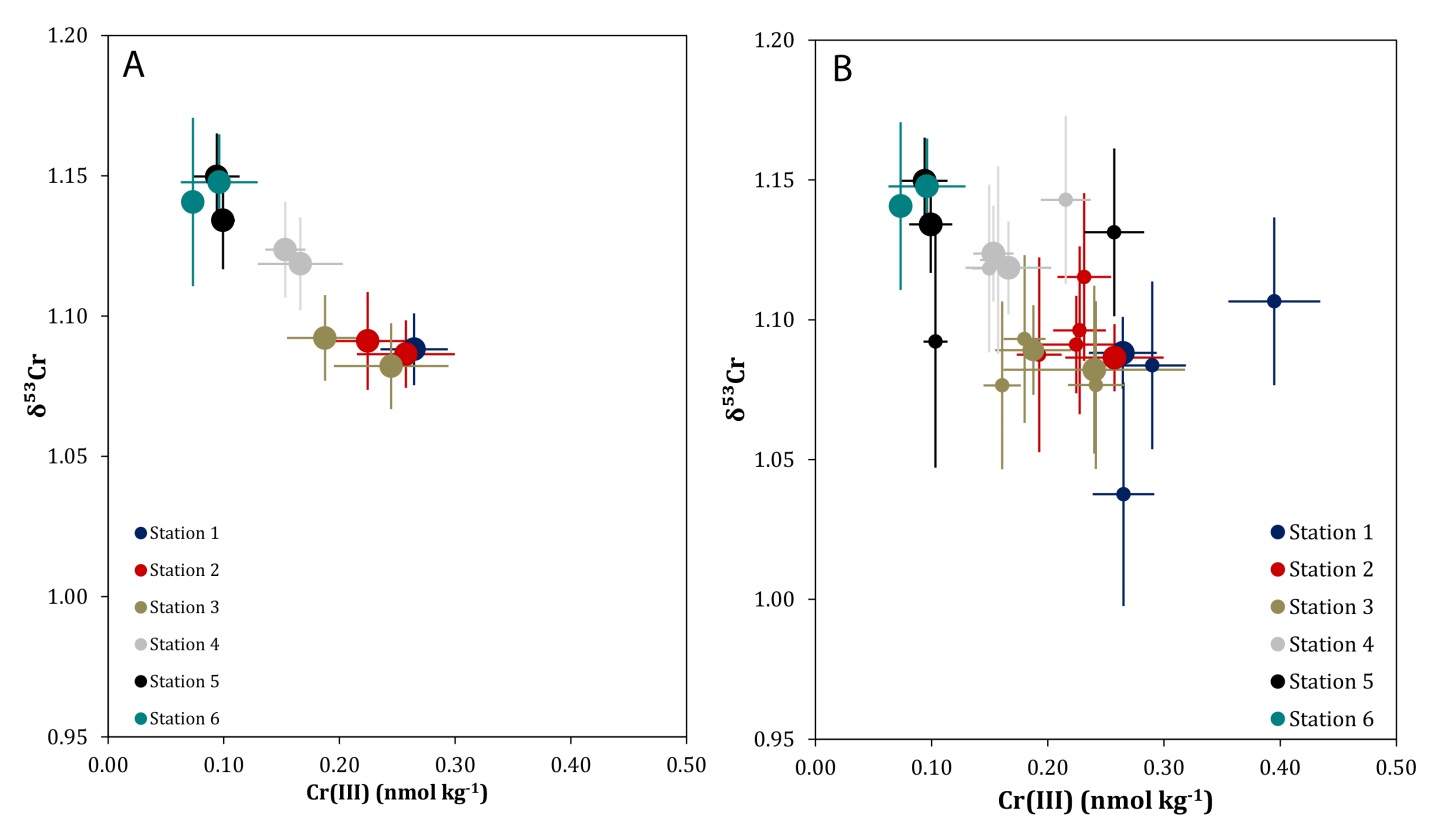


**Figure S.2: δ^53^Cr and [Cr(III)] data in the upper 100 m.** Panel (a) is analogous to Figure 4. All data from the upper 100 m are shown in panel (b), with larger circles representing data for which replicate Cr(III) samples are available.

| **Station** | **Cast** | **Depth** | **Neutral Density** | **Salinity** | **Potential Temperature** | **NO₃ + NO₂** | **Cr** | **Cr** | **δ^53^Cr** | **2 SEM** | **Cr(III)** | **Cr(III) error** | **Cr(III)** | **Cr(III) error** | **Cr(VI) + CR(III)_NR_** | **Cr(VI) + CR(III)_NR_ error** |
| --- | --- | --- | --- | --- | --- | --- | --- | --- | --- | --- | --- | --- | --- | --- | --- | --- |
|  |  | **m** | **kg m⁻³** |  | **°C** | **µM** | **ng kg^-1^** | **nmol kg^-1^** | **‰** | **‰** | **nmol kg^-1^** | **nmol kg^-1^** | **% total** | **% total** | **nmol/kg** | **nmol/kg** |
| 1 | **Mean** | 10 | 24.824 | 32.558 | 11.39 | 9.0 | **161** | **3.09** | **1.088** | **0.013** | **0.26** | **0.03** | **8.6** | **0.9** | **2.83** | **0.03** |
| 1 | 1 | 10 |  |  |  |  | 162 | 3.12 | 1.076 | 0.02 |  |  |  |  |  |  |
| 1 | 15 | 10 |  |  |  |  | 160 | 3.08 | 1.092 | 0.02 |  |  |  |  |  |  |
| 1 | 27 | 10 |  |  |  |  | 160 | 3.07 | 1.103 | 0.03 |  |  |  |  |  |  |
| 1 | 1 | 30 | 25.813 | 32.760 | 6.15 | 16.3 | 163 | 3.14 | 1.107 | 0.023 | 0.39 | 0.04 | 12.6 | 1.3 | 2.74 | 0.04 |
| 1 | 1 | 50 | 26.130 | 32.876 | 4.41 | 21.2 | 165 | 3.17 | 1.084 | 0.028 | 0.29 | 0.03 | 9.1 | 0.9 | 2.88 | 0.03 |
| 1 | 1 | 100 | 26.773 | 33.624 | 4.42 | 36.0 | 168 | 3.24 | 1.038 | 0.040 | 0.27 | 0.03 | 8.2 | 0.8 | 2.97 | 0.03 |
| 1 | 1 | 150 | 27.006 | 33.828 | 3.99 | 41.2 | 177 | 3.40 | 1.037 | 0.036 | 0.29 | 0.03 | 8.5 | 0.9 | 3.11 | 0.03 |
| 1 | 1 | 200 | 27.068 | 33.890 | 3.93 | 42.6 | 180 | 3.46 | 1.048 | 0.029 | 0.51 | 0.05 | 14.6 | 1.5 | 2.95 | 0.05 |
|  | | | | | | | | | | | | | | | | |
| 2 | **Mean** | 10 | 24.318 | 32.351 | 13.18 | 6.9 | **158** | **3.03** | **1.086** | **0.012** | **0.26** | **0.04** | **8.5** | **1.4** | **2.78** | **0.04** |
|  | 5 |  |  |  |  |  | 157 | 3.01 | 1.067 | 0.025 |  |  |  |  |  |  |
|  | 17 |  |  |  |  |  | 157 | 3.02 | 1.101 | 0.020 |  |  |  |  |  |  |
|  | 31 |  |  |  |  |  | 159 | 3.07 | 1.086 | 0.019 |  |  |  |  |  |  |
| 2 | 5 | 30 | 24.585 | 32.381 | 11.37 | 8.0 | 159 | 3.07 | 1.115 | 0.029 | 0.23 | 0.02 | 7.5 | 0.8 | 2.84 | 0.02 |
| 2 | **Mean** | 50 | 25.692 | 32.612 | 5.92 | 14.7 | **160** | **3.08** | **1.091** | **0.017** | **0.22** | **0.03** | **7.3** | **1.1** | **2.86** | **0.03** |
|  | 5 |  |  |  |  |  | 160 | 3.07 | 1.094 | 0.023 |  |  |  |  |  |  |
|  | 27 |  |  |  |  |  | 161 | 3.10 | 1.087 | 0.026 |  |  |  |  |  |  |
| 2 | 5 | 75 | 25.871 | 32.612 | 4.77 | 16.9 | 162 | 3.11 | 1.096 | 0.025 | 0.23 | 0.02 | 7.3 | 0.7 | 2.88 | 0.02 |
| 2 | 5 | 100 | 25.963 | 32.667 | 4.37 | 19.2 | 162 | 3.11 | 1.087 | 0.035 | 0.19 | 0.02 | 6.2 | 0.6 | 2.92 | 0.02 |
| 2 | 5 | 150 | 26.740 | 33.799 | 5.84 | 26.9 | 177* | 3.40* | 0.966* | 0.033* | 0.20 | 0.02 | NA | NA | NA | NA |
| 2 | 5 | 200 | 26.810 | 33.828 | 5.53 | 29.5 | 165 | 3.17 | 1.076 | 0.027 | 0.20 | 0.02 | 6.1 | 0.6 | 2.98 | 0.02 |
|  | | | | | | | | | | | | | | | | |
| 3 | **Mean** | 10 | 24.350 | 32.443 | 13.38 | 6.6 | **160** | **3.08** | **1.092** | **0.015** | **0.19** | **0.03** | **6.1** | **1.1** | **2.89** | **0.03** |
|  | 1 |  |  |  |  |  | 163 | 3.14 | 1.121 | 0.049 |  |  |  |  |  |  |
|  | 20 |  |  |  |  |  | 159 | 3.06 | 1.080 | 0.021 |  |  |  |  |  |  |
|  | 31 |  |  |  |  |  | 158 | 3.03 | 1.100 | 0.024 |  |  |  |  |  |  |
| 3 | 1 | 30 | 24.350 | 32.439 | 13.37 | 6.90 | 161 | 3.10 | 1.077 | 0.027 | 0.24 | 0.02 | 7.8 | 0.8 | 2.86 | 0.02 |
| 3 | 1 | 50 | 25.485 | 32.632 | 7.78 | 13.3 | 163 | 3.14 | 1.093 | 0.027 | 0.18 | 0.02 | 5.7 | 0.6 | 2.96 | 0.02 |
| 3 | 1-15 | 75 | 25.719¹ | 32.714¹ | 6.62¹ | 15.3 | 162¹ | 3.12¹ | 1.082¹ | 0.015¹ | **0.24** | **0.05** | **7.8** | **1.6** | **2.88** | **0.05** |
|  | 18-32 |  | 25.721² | 32.753² | 6.84² | 12.8³ | NA | NA | NA | NA | **0.11** | **0.02** | NA | NA | NA | NA |
| 3 | 1 | 100 | 25.833 | 32.815 | 6.39 | 15.8 | 162 | 3.12 | 1.077 | 0.028 | 0.16 | 0.02 | 5.2 | 0.5 | 2.96 | 0.02 |
| 3 | 1 | 150 | 26.673 | 33.756 | 6.18 | 24.5 | 164 | 3.15 | 1.104 | 0.028 | 0.28 | 0.03 | 9.0 | 0.9 | 2.87 | 0.03 |
| 3 | 1 | 200 | 26.764 | 33.804 | 5.73 | 27.7 | 166 | 3.19 | 1.078 | 0.023 | **0.15** | **0.01** | **4.7** | **0.4** | **3.04** | **0.01** |
|  | | | | | | | | | | | | | | | | |
| 4 | **Mean** | 10 | 23.824 | 32.724 | 16.81 | 0.8 | **156** | **3.00** | **1.119** | **0.017** | **0.17** | **0.04** | **5.5** | **1.2** | **2.84** | **0.04** |
|  | 2 |  |  |  |  |  | 158 | 3.04 | 1.135 | 0.026 |  |  |  |  |  |  |
|  | 8 |  |  |  |  |  | 156 | 3.00 | 1.119 | 0.030 |  |  |  |  |  |  |
|  | 17 |  |  |  |  |  | 155 | 2.98 | 1.096 | 0.030 |  |  |  |  |  |  |
| 4 | 2 | 30 | 24.505 | 32.986 | 14.67 | 0.3 | 159 | 3.05 | 1.143 | 0.025 | 0.22 | 0.02 | 7.1 | 0.7 | 2.83 | 0.02 |
| 4 | **Mean** | 50 | 25.335 | 33.275 | 11.71 | 3.6 | **157** | **3.01** | **1.124** | **0.017** | **0.15** | **0.02** | **5.1** | **0.6** | **2.86** | **0.02** |
| 4 | 2 |  |  |  |  |  | 155 | 2.99 | 1.132 | 0.022 |  |  |  |  |  |  |
| 4 | 17 |  |  |  |  |  | 158 | 3.04 | 1.112 | 0.026 |  |  |  |  |  |  |
| 4 | 2 | 75 | 25.690 | 33.215 | 9.47 | 9.9 | 158 | 3.04 | 1.118 | 0.029 | 0.15 | 0.01 | 4.9 | 0.5 | 2.89 | 0.01 |
| 4 | 2 | 100 | 25.857 | 33.450 | 9.61 | 10.3 | 156 | 3.00 | 1.121 | 0.034 | 0.16 | 0.02 | 5.2 | 0.5 | 2.84 | 0.02 |
| 4 | 2 | 150 | 26.352 | 33.952 | 9.10 | 14.8 | 159 | 3.05 | 1.122 | 0.037 | 0.10 | 0.01 | 3.4 | 0.3 | 2.94 | 0.01 |
| 4 | 2 | 200 | 26.426 | 33.907 | 8.45 | 15.5 | 158 | 3.04 | 1.105 | 0.029 | 0.12 | 0.01 | 3.8 | 0.4 | 2.92 | 0.01 |
|  | | | | | | | | | | | | | | | | |
| 5 | **Mean** | 10 | 23.448 | 34.651 | 23.79 | 0.1 | **157** | **3.02** | **1.150** | **0.015** | **0.09** | **0.02** | **3.1** | **0.7** | **2.93** | **0.02** |
|  | 1 |  |  |  |  |  | 157 | 3.02 | 1.137 | 0.022 |  |  |  |  |  |  |
|  | 15 |  |  |  |  |  | 157 | 3.02 | 1.163 | 0.022 |  |  |  |  |  |  |
| 5 | 1 | 30 | 24.665 | 34.343 | 18.49 | 0.1 | 156 | 3.01 | 1.092 | 0.045 | 0.10 | 0.01 | 3.4 | 0.3 | 2.90 | 0.01 |
| 5 | 1 | 50 | 25.122 | 34.359 | 16.68 | 0.2 | NA | NA | NA | NA | 0.09 | 0.01 | NA | NA | NA | NA |
| 5 | 1 | 75 | 25.515 | 34.282 | 14.71 | 0.2 | 157 | 3.02 | 1.131 | 0.027 | 0.26 | 0.03 | 8.5 | 0.9 | 2.76 | 0.03 |
| 5 | **Mean** | 90 | 25.639 | 34.263 | 14.08 | NA | **158** | **3.05** | **1.134** | **0.017** | **0.10** | **0.02** | **3.3** | **0.6** | **2.95** | **0.02** |
|  | 1 |  |  |  |  |  | 157 | 3.02 | 1.130 | 0.032 |  |  |  |  |  |  |
|  | 15 |  |  |  |  |  | 160 | 3.08 | 1.136 | 0.021 |  |  |  |  |  |  |
| 5 | 1 | 150 | 25.954 | 34.286 | 12.68 | 9.1 | 161 | 3.09 | 1.066 | 0.041 | 0.08 | 0.01 | 2.6 | 0.3 | 3.01 | 0.01 |
| 5 | **Mean** | 200 | 26.114 | 34.232 | 11.67 | 10.9 | **160** | **3.07** | **1.131** | **0.016** | **0.09** | **0.01** | **2.8** | **0.4** | **2.99** | **0.01** |
|  | 1 |  |  |  |  |  | 157 | 3.03 | 1.129 | 0.016 |  |  |  |  |  |  |
|  | 15 |  |  |  |  |  | 162 | 3.12 | 1.161 | 0.078 |  |  |  |  |  |  |
| 5 | 15 | 300 | 26.283 | 34.151 | 10.45 | NA | 154 | 2.96 | 1.076 | 0.050 | 0.08 | 0.01 | 2.5 | 0.3 | 2.88 | 0.01 |
| 5 | 15 | 400 | 26.520 | 34.080 | 8.79 | NA | 151 | 2.90 | 1.047 | 0.040 | 0.13 | 0.01 | 4.5 | 0.5 | 2.77 | 0.01 |
| 5 | 15 | 600 | 26.985 | 34.010 | 5.42 | NA | 181 | 3.48 | 1.042 | 0.041 | 0.10 | 0.01 | 2.9 | 0.3 | 3.38 | 0.01 |
| 5 | 15 | 800 | 27.311 | 34.188 | 4.09 | NA | 193 | 3.70 | 0.956 | 0.058 | 0.15 | 0.01 | 4.0 | 0.4 | 3.55 | 0.01 |
| 5 | 9 | 1000 | 27.505 | 34.342 | 3.54 | NA | 199 | 3.83 | 0.938 | 0.038 | 0.12 | 0.01 | 3.1 | 0.3 | 3.71 | 0.01 |
|  | | | | | | | | | | | | | | | | |
| 6 | **Mean** | 10 | 22.508 | 35.003 | 27.67 | 0.0589 | **155** | **2.98** | **1.148** | **0.017** | **0.10** | **0.03** | **3.2** | **1.1** | **2.89** | **0.03** |
|  | 1 |  |  |  |  |  | 155 | 2.99 | 1.145 | 0.030 |  |  |  |  |  |  |
|  | 5 |  |  |  |  |  | 155 | 2.98 | 1.149 | 0.021 |  |  |  |  |  |  |
| 6 | 1 | 100 | 24.302 | 35.172 | 22.20 | 0.2 | 158 | 3.04 | 1.141 | 0.024 | **0.07** | **0.01** | **2.4** | **0.2** | **2.96** | **0.01** |

**Table S.1: All data**. Values in bold are means of replicates (δ^53^Cr = weighted mean, all others = arithmetic mean). δ^53^Cr data are shown with 2 SEM internal uncertainties for individual measurements or 2 SEM for the weighted mean of replicate measurements. Unless otherwise noted, CTD data are from the same cast as the Cr depth profile. Neutral density and potential temperature were calculated from CTD data using Ocean Data View (Schlitzer, 2018). Starting coordinates for sampling stations – Station 1: 54° 59.98’ N, 147° 12.95’ W; Station 2: 50° 0.10’ N, 144° 59.96’ W; Station 3: 46° 0.03’ N, 157° 59.96’ W; Station 4: 41° 59.83’ N, 158° 0.51’ W; Station 5: 33° 59.99’ N, 158° 0.00’ W; Station 6: 24° 10.03’ N, 160° 15.23’ W.

NA = data not available.

*Sample omitted from figures and discussion, contamination during processing

¹From station 3, cast 1

²From station 3, cast 18

³From station 3, cast 32.

|  | **Cr** | **δ^53^Cr** |  | **Cr** | **δ^53^Cr** |
| --- | --- | --- | --- | --- | --- |
|  | **nmol kg^-1^** | **‰** |  | **nmol kg^-1^** | **‰** |
| *osil 2 USask* | *4.38* | *0.68* | stn2-5-50 | 3.07 | 1.09 |
| *osil 2 USask* | *4.22* | *0.66* | stn2-27-50 | 3.10 | 1.09 |
| **Average** | **4.30** | **0.67** | **Average** | **3.08** | **1.09** |
| **1RSD ([Cr]), 2SD (δ^53^Cr)** | **2.7** | **0.036** | **1RSD ([Cr]), 2SD (δ^53^Cr)** | **0.6** | **0.010** |
| *osil16 USask* | *3.23* | *0.97* | stn3-20-10 | 3.06 | 1.08 |
| *osil16 USask* | *3.24* | *0.98* | stn3-31-10 | 3.03 | 1.10 |
| **Average** | **3.23** | **0.97** | **Average** | **3.05** | **1.09** |
| **1RSD ([Cr]), 2SD (δ^53^Cr)** | **0.1** | **0.004** | **1RSD ([Cr]), 2SD (δ^53^Cr)** | **0.7** | **0.028** |
| *Osil Bern bottle 1* | *4.03* | *0.85* | stn4-1-10 | 3.04 | 1.14 |
| *Osil Bern bottle 1* | *3.99* | *0.82* | stn4-8-10 | 3.00 | 1.12 |
| *Osil Bern bottle 1* | *4.05* | *0.86* | stn4-17-10 | 2.98 | 1.10 |
| **Average** | **4.02** | **0.84** | **Average** | **3.00** | **1.12** |
| **1RSD ([Cr]), 2SD (δ^53^Cr)** | **0.8** | **0.043** | **1RSD ([Cr]), 2SD (δ^53^Cr)** | **1.0** | **0.039** |
| *Osil Bern bottle 2* | *4.19* | *0.85* | stn4-1-50 | 2.99 | 1.13 |
| *Osil Bern bottle 2* | *4.18* | *0.83* | stn4-17-50 | 3.04 | 1.11 |
| *Osil Bern bottle 2* | 4.17 | 0.81 | **Average** | **3.01** | **1.12** |
| Osil Bern bottle 2 | 4.18 | 0.80 | **1RSD ([Cr]), 2SD (δ^53^Cr)** | **1.2** | **0.028** |
| Osil Bern bottle 2 | 4.16 | 0.81 | stn5-1-10 | 3.02 | 1.14 |
| **Average** | **4.18** | **0.82** | stn5-15-10 | 3.02 | 1.16 |
| **1RSD ([Cr]), 2SD (δ^53^Cr)** | **0.3** | **0.037** | **Average** | **3.02** | **1.15** |
| OSIL Bern bottle 3 | 4.18 | 0.83 | **1RSD ([Cr]), 2SD (δ^53^Cr)** | **0.0** | **0.037** |
| OSIL Bern bottle 3 | 4.20 | 0.83 | stn5-1-90 | 3.02 | 1.13 |
| OSIL Bern bottle 3 | 4.18 | 0.83 | stn5-15-90 | 3.08 | 1.14 |
| **Average** | **4.19** | **0.83** | **Average** | **3.05** | **1.13** |
| **1RSD ([Cr]), 2SD (δ^53^Cr)** | **0.2** | **0.002** | **1RSD ([Cr]), 2SD (δ^53^Cr)** | **1.4** | **0.008** |
| ANT 23/1 | 3.05 | 1.11 | stn 6-1-10 | 2.99 | 1.14 |
| ANT 23/1 | 3.05 | 1.11 | stn 6-5-10 | 2.98 | 1.15 |
| ANT 23/1 | 3.06 | 1.10 | **Average** | **2.98** | **1.15** |
| ANT 23/1 | 3.06 | 1.09 | **1RSD ([Cr]), 2SD (δ^53^Cr)** | **0.1** | **0.007** |
| ANT 23/1 | 3.06 | 1.12 | *tm9-50* | *3.44* | *0.95* |
| ANT 23/1 | 3.08 | 1.08 | *tm9-50 rep* | *3.44* | *0.99* |
| ANT 23/1 | 3.09 | 1.09 | **Average** | **3.44** | **0.97** |
| ANT 23/1 | 3.10 | 1.08 | **1RSD ([Cr]), 2SD (δ^53^Cr)** | **0.0** | **0.054** |
| ANT 23/1 | 3.09 | 1.06 | *tm10-80* | *3.75* | *0.91* |
| **Average** | **3.07** | **1.09** | *tm10-80 rep* | *3.73* | *0.92* |
| **1RSD ([Cr]), 2SD (δ^53^Cr)** | **0.6** | **0.037** | **Average** | **3.74** | **0.91** |
| stn1-1-10 | 3.12 | 1.076 | **1RSD ([Cr]), 2SD (δ^53^Cr)** | **0.2** | **0.011** |
| stn1-15-10 | 3.08 | 1.092 |  |  |  |
| stn1-27-10 | 3.07 | 1.103 | **Summary** | | |
| **Average** | **3.09** | **1.09** |  |  |  |
| **1RSD ([Cr]), 2SD (δ^53^Cr)** | **0.9** | **0.027** |  | **Cr (nmol kg^-1^)** | **δ^53^Cr (‰)** |
| stn2-5-10 | 3.01 | 1.067 | **Measurements** | 49 | 49 |
| stn2-17-10 | 3.02 | 1.101 | **Samples** | 17 | 17 |
| stn2-31-10 | 3.07 | 1.086 |  | 1RSD (%) | 2SD (‰) |
| **Average** | **3.03** | **1.08** | **Error** | **0.82** | **0.0328** |
| **1RSD ([Cr]), 2SD (δ^53^Cr)** | **0.9** | **0.034** |  |  |  |

**Table S.2: All replicate seawater analyses used to assess external reproducibility**. Individual analyses are shown with sample average values.

| **Station** | **Cast** | **Time** | **Depth** | **Cr(III)** | **Stdev** | **Depth** | **Cr(III)** | **Stdev** | **Depth** | **Cr(III)** | **Stdev** |  |  |
| --- | --- | --- | --- | --- | --- | --- | --- | --- | --- | --- | --- | --- | --- |
|  |  | local | m | nmol kg^-1^ | nmol kg^-1^ | m | nmol kg^-1^ | nmol kg^-1^ | m | nmol kg^-1^ | nmol kg^-1^ |  |  |
| **1** | **1** | **14:15** | *10 A* | *0.42* | *0.04* | NA | | | NA | | |  |  |
| **1** | **1** | **14:15** | 10 B | 0.27 | 0.03 | NA | | | NA | | |  |  |
| **1** | **1** | **14:15** | 10 C | 0.28 | 0.03 | NA | | | NA | | |  |  |
| **1** | **8** | **01:35** | 10 A | 0.24 | 0.02 | NA | | | NA | | |  |  |
| **1** | **8** | **01:35** | 10 B | 0.26 | 0.03 | NA | | | NA | | |  |  |
| **1** | **8** | **01:35** | 10 C | 0.24 | 0.02 | NA | | | NA | | |  |  |
| **1** | **12** | **07:40** | 10 A | 0.24 | 0.02 | NA | | | NA | | |  |  |
| **1** | **12** | **07:40** | 10 B | 0.22 | 0.02 | NA | | | NA | | |  |  |
| **1** | **12** | **07:40** | 10 C | 0.24 | 0.02 | NA | | | NA | | |  |  |
| **1** | **15** | **13:35** | 10 A | 0.27 | 0.03 | NA | | | NA | | |  |  |
| **1** | **15** | **13:35** | 10 B | 0.28 | 0.03 | NA | | | NA | | |  |  |
| **1** | **15** | **13:35** | 10 C | 0.28 | 0.03 | NA | | | NA | | |  |  |
| **1** | **20** | **19:38** | 10 A | 0.28 | 0.03 | NA | | | NA | | |  |  |
| **1** | **20** | **19:38** | 10 B | 0.34 | 0.03 | NA | | | NA | | |  |  |
| **1** | **20** | **19:38** | 10 C | 0.22 | 0.02 | NA | | | NA | | |  |  |
| **1** | **24** | **01:35** | *10 A* | *0.55* | *0.06* | NA | | | NA | | |  |  |
| **1** | **24** | **01:35** | *10 B* | *0.35* | *0.04* | NA | | | NA | | |  |  |
| **1** | **24** | **01:35** | *10 C* | *0.37* | *0.04* | NA | | | NA | | |  |  |
| **1** | **27** | **07:30** | 10 A | 0.29 | 0.03 | NA | | | NA | | |  |  |
| **1** | **27** | **07:30** | 10 B | 0.26 | 0.03 | NA | | | NA | | |  |  |
| **1** | **27** | **07:30** | 10 C | 0.29 | 0.03 | NA | | | NA | | |  |  |
| **Station** | **Cast** | **Time** | **Depth** | **Cr(III)** | **Stdev** | **Depth** | **Cr(III)** | **Stdev** | **Depth** | **Cr(III)** | **Stdev** |  |  |
|  |  | local | m | nmol kg^-1^ | nmol kg^-1^ | m | nmol kg^-1^ | nmol kg^-1^ | m | nmol kg^-1^ | nmol kg^-1^ |  |  |
| **2** | **1** | **01:45** | 10 | 0.25 | 0.02 | 50 | 0.18 | 0.02 | NA | | |  |  |
| **2** | **5** | **06:00** | 10 A | 0.21 | 0.02 | 50 A | 0.28 | 0.03 | NA | | |  |  |
| **2** | **5** | **06:00** | 10 B | 0.27 | 0.03 | 50 B | 0.27 | 0.03 | NA | | |  |  |
| **2** | **7** | **09:40** | 10 | 0.30 | 0.03 | 50 | 0.20 | 0.02 | NA | | |  |  |
| **2** | **9** | **13:30** | 10 | 0.33 | 0.03 | 50 | 0.22 | 0.02 | NA | | |  |  |
| **2** | **13** | **17:55** | 10 | 0.21 | 0.02 | 50 | 0.22 | 0.02 | NA | | |  |  |
| **2** | **15** | **21:27** | 10 | 0.32 | 0.03 | 50 | 0.23 | 0.02 | NA | | |  |  |
| **2** | **17** | **01:45** | 10 | 0.23 | 0.02 | 50 | 0.22 | 0.02 | NA | | |  |  |
| **2** | **19** | **05:30** | 10 | 0.23 | 0.02 | 50 | 0.23 | 0.02 | NA | | |  |  |
| **2** | **22** | **09:35** | 10 A | 0.26 | 0.03 | *50* | *0.37* | *0.04* | NA | | |  |  |
| **2** | **22** | **09:35** | 10 B | 0.26 | 0.03 | NA | | | NA | | |  | NA |
| **2** | **24** | **13:27** | 10 | 0.29 | 0.03 | 50 | 0.19 | 0.02 | NA | | |  |  |
| **2** | **27** | **17:45** | 10 | 0.20 | 0.02 | 50 | 0.26 | 0.03 | NA | | |  |  |
| **2** | **29** | **21:28** | 10 | 0.26 | 0.03 | 50 | 0.16 | 0.02 | NA | | |  |  |
| **2** | **31** | **01:35** | 10 | 0.19 | 0.02 | 50 | 0.21 | 0.02 | NA | | |  |  |
| **Station** | **Cast** | **Time** | **Depth** | **Cr(III)** | **Stdev** | **Depth** | **Cr(III)** | **Stdev** | **Depth** | **Cr(III)** | **Stdev** |  |  |
|  |  | local | m | nmol kg^-1^ | nmol kg^-1^ | m | nmol kg^-1^ | nmol kg^-1^ | m | nmol kg^-1^ | nmol kg^-1^ |  |  |
| **3** | **1** | **07:25** | *10 A* | *0.31* | *0.03* | 75 A | 0.29 | 0.03 | 200 | 0.13 | 0.01 |  |  |
| **3** | **1** | **07:25** | 10 B | 0.16 | 0.02 | 75 B | 0.19 | 0.02 | NA | | |  |  |
| **3** | **4** | **09:45** | 10 | 0.22 | 0.02 | 75 | 0.29 | 0.03 | 200 | 0.15 | 0.01 |  |  |
| **3** | **6** | **13:35** | 10 | 0.18 | 0.02 | 75 | 0.25 | 0.03 | 200 | 0.13 | 0.01 |  |  |
| **3** | **11** | **17:35** | 10 | 0.21 | 0.02 | 75 | 0.29 | 0.03 | 200 | 0.17 | 0.02 |  |  |
| **3** | **13** | **21:25** | 10 | 0.22 | 0.02 | 75 | 0.27 | 0.03 | *200* | 0.23 | *0.02* |  |  |
| **3** | **15** | **01:31** | 10 | 0.22 | 0.02 | 75 | 0.17 | 0.02 | 200 | 0.16 | 0.02 |  |  |
| **3** | **18** | **05:24** | 10 A | 0.22 | 0.02 | 75 A | 0.21 | 0.02 | *200 A* | *0.30* | *0.03* |  |  |
| **3** | **18** | **05:24** | 10 B | 0.19 | 0.02 | 75 B | 0.13 | 0.01 | 200 B | 0.16 | 0.02 |  |  |
| **3** | **20** | **09:25** | 10 | 0.15 | 0.02 | 75 | 0.13 | 0.01 | NA | | |  |  |
| **3** | **23** | **13:27** | 10 | 0.14 | 0.01 | 75 | 0.11 | 0.01 | NA | | |  |  |
| **3** | **25** | **17:25** | 10 | 0.13 | 0.01 | 75 | 0.10 | 0.01 | NA | | |  |  |
| **3** | **27** | **21:20** | 10 | 0.17 | 0.02 | 75 | 0.13 | 0.01 | NA | | |  |  |
| **3** | **29** | **01:25** | *10* | *0.26* | *0.03* | 75 | 0.09 | 0.01 | NA | | |  |  |
| **3** | **31** | **05:30** | 10 | 0.20 | 0.02 | *75* | *0.57* | *0.06* | NA | | |  |  |
| **Station** | **Cast** | **Time** | **Depth** | **Cr(III)** | **Stdev** | **Depth** | **Cr(III)** | **Stdev** | **Depth** | **Cr(III)** | **Stdev** |  |  |
|  |  | local | m | nmol kg^-1^ | nmol kg^-1^ | m | nmol kg^-1^ | nmol kg^-1^ | m | nmol kg^-1^ | nmol kg^-1^ |  |  |
| **4** | **2** | **09:20** | 10 A | 0.18 | 0.02 | 50 | 0.18 | 0.02 | NA | | |  |  |
| **4** | **2** | **09:20** | 10 B | 0.23 | 0.02 | NA | | | NA | | |  |  |
| **4** | **4** | **13:20** | 10 | 0.17 | 0.02 | 50 | 0.17 | 0.02 | NA | | |  |  |
| **4** | **8** | **17:30** | *10* | *0.27* | *0.03* | 50 | 0.13 | 0.01 | NA | | |  |  |
| **4** | **10** | **21:22** | 10 | 0.16 | 0.02 | 50 | 0.15 | 0.02 | NA | | |  |  |
| **4** | **12** | **01:20** | 10 | 0.21 | 0.02 | 50 | 0.17 | 0.02 | NA | | |  |  |
| **4** | **15** | **05:38** | 10 | 0.18 | 0.02 | *50* | *0.26* | *0.03* | NA | | |  |  |
| **4** | **17** | **09:25** | 10 | 0.19 | 0.02 | 50 | 0.17 | 0.02 | NA | | |  |  |
| **4** | **20** | **13:20** | 10 | 0.14 | 0.01 | 50 | 0.16 | 0.02 | NA | | |  |  |
| **4** | **22** | **17:28** | 10 | 0.15 | 0.02 | 50 | 0.14 | 0.01 | NA | | |  |  |
| **4** | **24** | **21:25** | 10 | 0.15 | 0.01 | 50 | 0.14 | 0.01 | NA | | |  |  |
| **4** | **26** | **01:32** | 10 | 0.11 | 0.01 | *50* | *0.39* | *0.04* | NA | | |  |  |
| **4** | **28** | **05:27** | 10 | 0.11 | 0.01 | 50 | 0.13 | 0.01 | NA | | |  |  |
| **Station** | **Cast** | **Time** | **Depth** | **Cr(III)** | **Stdev** | **Depth** | **Cr(III)** | **Stdev** | **Depth** | **Cr(III)** | **Stdev** |  |  |
|  |  | local | m | nmol kg^-1^ | nmol kg^-1^ | m | nmol kg^-1^ | nmol kg^-1^ | m | nmol kg^-1^ | nmol kg^-1^ |  |  |
| **5** | **1** | **03:50** | 10 | 0.11 | 0.01 | *90 A* | *0.32* | *0.03* | 200 | 0.07 | 0.01 |  |  |
| **5** | **1** | **03:50** | NA | | | 90 B | 0.10 | 0.01 | NA | | |  |  |
| **5** | **4** | **09:25** | 10 | 0.11 | 0.01 | NA | | | 200 | 0.10 | 0.01 |  |  |
| **5** | **7** | **11:34** | 10 A | 0.08 | 0.01 | 90 A | 0.09 | 0.01 | NA | | |  |  |
| **5** | **7** | **11:34** | 10 B | 0.06 | 0.01 | 90 B | 0.07 | 0.01 | NA | | |  |  |
| **5** | **9** | **15:00** | *10* | 0.21 | *0.02* | 90 | 0.09 | 0.01 | 200 | 0.10 | 0.01 |  |  |
| **5** | **11** | **17:23** | 10 | 0.10 | 0.01 | *90* | *0.20* | *0.02* | 200 | 0.09 | 0.01 |  |  |
| **5** | **13** | **21:20** | 10 | 0.07 | 0.01 | 90 | 0.13 | 0.01 | 200 | 0.08 | 0.01 |  |  |
| **5** | **15** | **02:00** | 10 | 0.08 | 0.01 | 90 | 0.11 | 0.01 | NA | | |  |  |
| **5** | **18** | **05:32** | *10* | *0.84* | *0.08* | 90 | 0.08 | 0.01 | NA | | |  |  |
| **5** | **20** | **09:25** | 10 | 0.11 | 0.01 | 90 | 0.13 | 0.01 | NA | | |  |  |
| **5** | **24** | **15:22** | *10* | *0.17* | *0.02* | 90 | 0.09 | 0.01 | NA | | |  |  |
| **5** | **27** | **21:24** | 10 | 0.12 | 0.01 | 90 | 0.10 | 0.01 | NA | | |  |  |
| **5** | **32** | **05:30** | 10 | 0.10 | 0.01 | *90* | *0.26* | *0.03* | NA | | |  |  |
| **Station** | **Cast** | **Time** | **Depth** | **Cr(III)** | **Stdev** | **Depth** | **Cr(III)** | **Stdev** | **Depth** | **Cr(III)** | **Stdev** |  |  |
|  |  | local | m | nmol kg^-1^ | nmol kg^-1^ | m | nmol kg^-1^ | nmol kg^-1^ | m | nmol kg^-1^ | nmol kg^-1^ |  |  |
| **6** | **1** | **05:55** | 10 | 0.07 | 0.01 | 100 | 0.07 | 0.01 | NA | | |  |  |
| **6** | **3** | **11:15** | NA | | | 100 | 0.08 | 0.01 | NA | | |  | NA |
| **6** | **5** | **15:15** | 10 | 0.10 | 0.01 | 100 | 0.08 | 0.01 | NA | | |  |  |
| **6** | **7** | **19:20** | 10 | 0.15 | 0.01 | 100 | 0.08 | 0.01 | NA | | |  |  |
| **6** | **9** | **23:20** | 10 | 0.09 | 0.01 | 100 | 0.07 | 0.01 | NA | | |  |  |
| **6** | **11** | **03:18** | 10 | 0.06 | 0.01 | 100 | 0.07 | 0.01 | NA | | |  |  |

**Table S.3 Diel Cr(III) cycle data**. All diel Cr(III) cycle data are shown, arranged by station, cast and local time of day. Samples were collected at 1 (Station 1), 2 (Stations 2, 4, 6) or 3 (Stations 3 and 5) depths per station. NA denotes that a sample for this depth and time point is not available.

| Station 1 | 45.7 ± 15.9 m |
| --- | --- |
| Station 2 | 41.8 ±7.8 m |
| Station 3 | 57.1 ± 17.0 m |
| Station 4 | 60.8 ± 4.0 m |
| Station 5 | 106.4 ± 21.1 m |
| Station 6 | 129.7 ± 20.7 m |

**Table S.4: Euphotic zone depths**.

Euphotic zones were determined as the 1% isolume, with 1 standard deviation based on all daylight casts while on station.

**References**

Janssen, D.J., Rickli, J., Quay, P.D., White, A.E., Nasemann, P., Jaccard, S.L. (2019) Seawater dissolved chromium concentration, redox speciation, and stable isotope composition in the North Pacific Ocean. (Version 1.0) [Data set]. Zenodo. http://doi.org/10.5281/zenodo.3560082

Schlitzer, R., (2018). Ocean Data View. Available at http://odv.awi.de.
